# Single‐cell multi‐omics deciphers hepatocyte dedifferentiation and illuminates maintenance strategies

**DOI:** 10.1111/cpr.13772

**Published:** 2025-01-14

**Authors:** Jie Hao, Zhenyi Wang, Jilong Ren, Shenghao Cao, Zhongchen Xie, Jinghuan Yang, Jiachen Li, Weizhe Ding, Jie Li, Zhiqiang Han, Ye Yuan, Tang Hai, Sheng Ding, Michael Q. Zhang, Minglei Shi

**Affiliations:** ^1^ School of Pharmaceutical Sciences Tsinghua University Beijing China; ^2^ MOE Key Laboratory of Bioinformatics, Beijing National Research Center for Information Science and Technology, Bioinformatics Division Tsinghua University Beijing China; ^3^ Shanghai Institute of Hematology, State Key Laboratory of Medical Genomics, National Research Center for Translational Medicine (Shanghai) Ruijin Hospital Affiliated to Shanghai Jiao Tong University School of Medicine Shanghai China; ^4^ Key Laboratory of Organ Regeneration and Reconstruction State Key Laboratory of Stem Cell and Reproductive Biology Institute of Zoology Chinese Academy of Sciences Beijing China; ^5^ Beijing Farm Animal Research Center, Institute of Zoology Chinese Academy of Sciences Beijing China; ^6^ Institute of Image Processing and Pattern Recognition Shanghai Jiao Tong University Shanghai China; ^7^ Key Laboratory of System Control and Information Processing Ministry of Education of China Shanghai China; ^8^ School of Life Sciences Tsinghua University Beijing China; ^9^ State Key Laboratory of Biopharmaceutical Preparation and Delivery Institute of Process Engineering Chinese Academy of Sciences Beijing China; ^10^ Department of Biological Sciences, Center for Systems Biology The University of Texas Richardson Texas USA

## Abstract

Due to the similarity to human hepatocytes, porcine hepatocytes play an important role in hepatic research and drug evaluation. However, once hepatocytes were cultured in vitro, it was often prone to dedifferentiate, resulting in the loss of their characteristic features and normal functions, which impede their application in liver transplantation and hepatotoxic drugs evaluation. Up to now, this process has yet to be thoroughly investigated from the single‐cell resolution and multi‐omics perspective. In this study, we utilized 10× multiome technology to dissect the heterogeneity of porcine hepatocytes at different time points (Days 0, 1, 3, 5 and 7) during dedifferentiation. We comprehensively investigated cell heterogeneity, cellular dynamics, signalling pathways, potential gene targets, enhancer‐driven gene regulatory networks, cell–cell communications of these cells and the conservation of mechanisms across species. We found that a series of critical signalling pathways driven by ERK, PI3K, Src and TGF‐β were activated during this process, especially in the early stage of dedifferentiation. Based on these discoveries, we constructed a chemical combination targeting these pathways, which effectively inhibited the dedifferentiation of porcine hepatocytes in vitro. To validate the effectiveness of this combination, we transplanted such treated hepatocytes into FRGN mice, and the results demonstrated that these cells could effectively repopulate the liver and improve the survival of mice.

## Graphical abstract

Primary porcine hepatocytes (PPHs) are important for hepatic research and clinical applications. With 10× multiome, we studied their dynamics during dedifferentiation and identified a series of key regulators of this process. Based on these findings, we further formulated a chemical combination that effectively prevents PPH dedifferentiation. The treated PPHs were validated with successful FRGN repopulation.

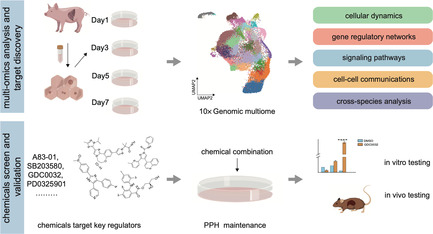

## 1 INTRODUCTION

Primary hepatocytes are the major cell type of the liver and perform a multitude of physiological functions, including albumin secretion, glycogen storage, drug metabolism and detoxification. However, the limited availability and variability between batches of human liver samples constrained their widespread application, so animal hepatocytes emerge as a viable alternative. Since the metabolic pathways of porcine hepatocytes closely resemble those of humans,^1^ they are viewed as a promising resource to substitute primary human hepatocytes in modelling metabolic diseases,^2^, ^3^ evaluating drug metabolism and toxicity^4^ and developing bioartificial liver devices.^5^, ^6^ Recently, the potential of primary porcine hepatocytes (PPHs) in basic research and clinical applications is increasingly recognized. For instance, Nature magazine^7^ reported the significant progress of xenotransplantation technology that employed pig livers for human liver transplantation. This breakthrough further underscores the growing importance of PPH in cutting‐edge medical research and potential clinical applications.

In many basic and clinical applications, hepatocytes need to be isolated from the body and cultured in vitro. It is crucial for primary hepatocytes to retain their original characteristics and functions during in vitro culture. However, primary hepatocytes often lose their identity and functions once they are removed from their native environment.^8^, ^9^ The mechanisms behind this phenomenon are not fully elucidated, and efforts to effectively control this process have not been entirely successful. Particularly, this issue is exacerbated for PPH, as research in this area is still scarce.

To understand this process, many studies have been carried out to dissect dedifferentiation at the level of bulk RNA‐seq,^10^, ^11^ transposase‐accessible chromatin using sequencing (ATAC‐seq)^11^ or single‐cell RNA‐seq (scRNA‐seq).^3^, ^12^ However, due to the inherent limitations of unimodal (mono‐omics), the classification of cell clusters based solely on mono‐omics lacks precision. The cell heterogeneity, cellular dynamics, signalling pathways, potential gene targets and enhancer‐driven gene regulatory networks (eGRNs) during the hepatic dedifferentiation process were still not fully understood.

Advances in sequencing technologies enabled the simultaneous measurement of ATAC‐seq and RNA‐seq in a single cell to analyse the regulation mechanism. Through the joint research of single cell and multi‐omics, it was possible to achieve more accurate cell identification,^13^ mutual validation of gene expression and chromatin accessibility, clear identification of cell type‐specific cis‐gene regulatory elements and transcription factors (TFs)^14^, ^15^ and explorations of cell type‐specific transcription regulation mechanisms.^16^, ^17^, ^18^

**FIGURE 1:**
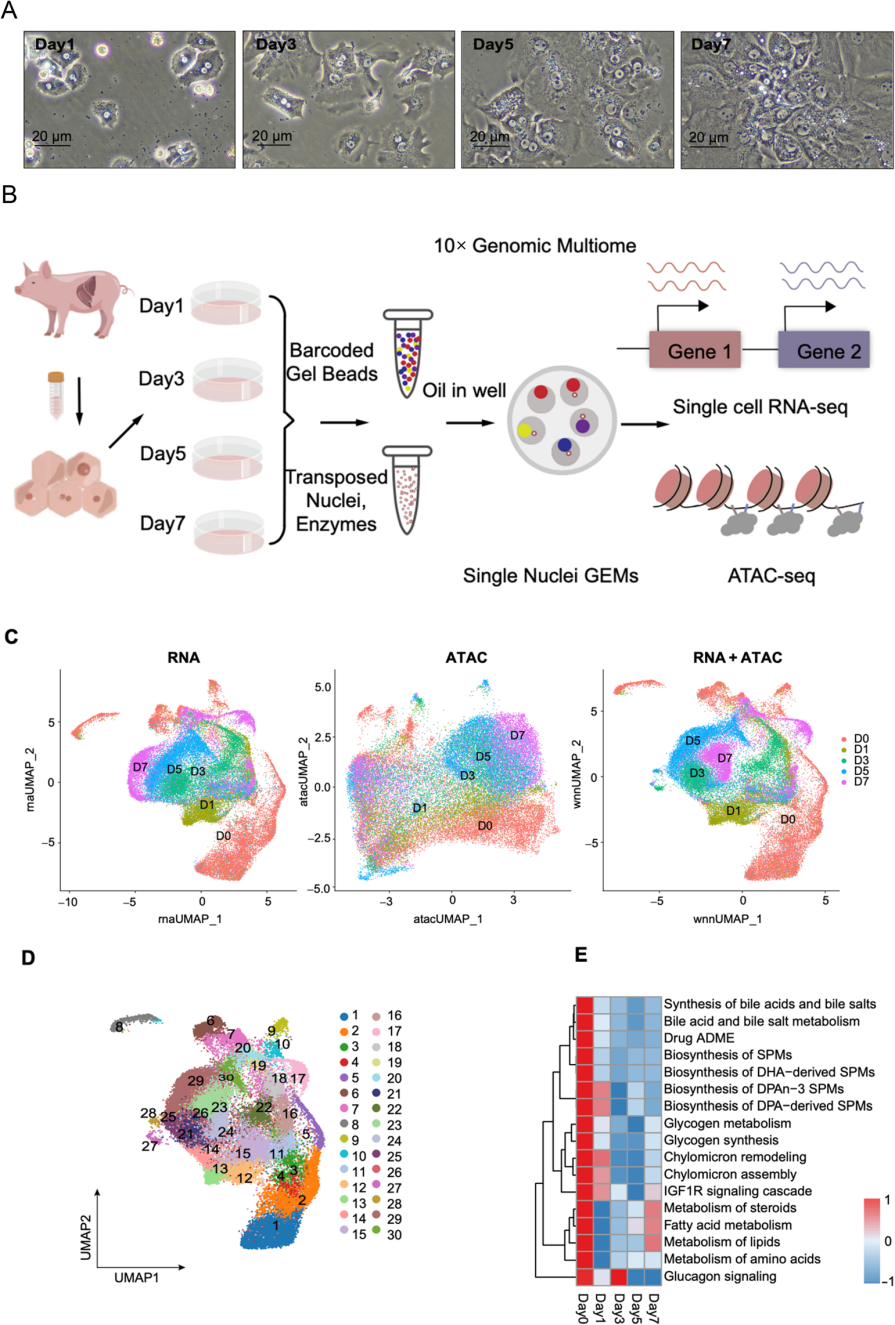
Primary porcine hepatocytes lose their mature phenotype during culture. (A) State of culturing liver cells in vitro for 1, 3, 5 and 7 days. (B) Experimental workflow demonstrating the application of Multiome technology for single‐cell sequencing. (C) Uniform manifold approximation and projection (UMAP) visualization of all cells according to their respective days of origin using single‐cell RNA and scATAC, individually and integrating. (D) UMAP plot showing the cell cluster after scaling using multi‐omics single‐cell data. (E) Heatmap showing the changes of GSVA enrichment in liver‐related pathways at different culture days. ATAC, transposase‐accessible chromatin.

In this study, we first utilized 10× multiome technology to construct a time‐series dataset of cultured PPH in vitro at Day 1 (D1), Day 3 (D3), Day 5 (D5) and Day 7 (D7) (Figure 1A,B). As 10× multiome can obtain the transcriptome and chromatin accessibility in a single cell, our approach provided precise cell identity, uncovered the TFs and signalling pathways modulating the hepatic dedifferentiation and led to the discovery of several potential targets. Subsequently, we conducted a screening of pertinent small molecules targeting these identified pathways and successfully constructed a chemical combination to block hepatic dedifferentiation in vitro. These findings offer new insights and potential strategies for maintaining hepatocyte functionality in vitro, which is crucial for advancements in hepatocyte‐based therapies and research.

## 2 MATERIALS AND METHODS

### 2.1 The isolation of PPH

PPHs were isolated from 6‐month‐old male Parma miniature pigs using a modified two‐step collagenase perfusion method.^19^ Briefly, intrahepatic blood was rinsed with Ca^2+^, Mg^2+^ ion‐free HBSS (containing 0.05 M HEPES, 10 mM EGTA) solution, and the liver was perfused with collagenase for liver digestion when the liver turned pale in colour. The mixed cells were repeatedly washed and centrifuged at 50*g* two times with Hepatocyte Wash Medium (17704024, Gibco, USA) to collect hepatocytes.

### 2.2 The plating and culture of PPH

For cell culture, 1 × 10^5^ cells were seeded in each well of a 12‐well plate and cultured in HCM medium (CC‐3198, Lonza). The medium was changed every other day. For chemical compound testing, each small molecule was added according to its concentration, and samples were collected after a 5‐day treatment.

### 2.3 Nuclei preparation and 10× multiome sequencing

Nuclei were prepared according to the 10× Genomics protocol (CG000365 • Rev. C), with the following modifications: The hepatocyte pellets were resuspended in 100 μL chilled lysis buffer and incubated on ice for 10 min. After adding 1 mL of chilled wash buffer, nuclei were centrifuged at 500*g* for 5 min at 4°C. The nuclear pellets were then washed with 45 μL chilled diluted nuclei buffer without pipetting. Joint scRNA‐seq and scATAC‐seq libraries were prepared using the 10× Genomics Single Cell Multiome ATAC+ Gene Expression kit (CG000338 Rev. E). Libraries were sequenced on an Illumina NovaSeq 6000 and an Illumina NextSeq 550AR to target depths of 300 and 150 million read pairs, respectively.

### 2.4 Data processing and quality control

The computational environment used in this research was CentOS Linux 7 (Core), and the program languages were R (v.4.1.1) and Python (v.3.6.7).

For 10× multiome data, the raw single‐cell multiome sequencing data were demultiplexed and aligned to the Sus scrofa ensemble reference (v.11.1) using Cell Ranger‐arc (v.2.0.1). Quality control was performed by combining information from scRNA‐seq and scATAC‐seq data. In our dataset, each day's data is a sequencing batch. To accurately filter out low‐quality data, we performed quality control on each day's data separately before merging them. First, low‐quality cells were filtered out by applying specific criteria to both scRNA‐seq and scATAC‐seq data. It retains cells where the scATAC‐seq read count is between 400 and 100,000, the scRNA‐seq read count is between 300 and 30,000, and the percentage of mitochondrial RNA is less than 32. Cells that do not meet these criteria were excluded. Then, we utilized ‘CreateChromatinAssay’ function in Signac (v.1.3.0) to include features of the scATAC‐seq matrix detected in at least 10 cells. For scRNA‐seq data, the read count matrix undergoes normalization and transformation using the SCTransform method, followed by principal component analysis (PCA) and uniform manifold approximation and projection (UMAP) on the first 50 principal components. For the scATAC‐seq data, Term Frequency‐Inverse Document Frequency normalization is applied, and top features are identified by ‘FindTopFeatures’ function. Singular value decomposition is performed, excluding the first dimension which typically represents sequencing depth, followed by UMAP on the second to the fiftieth dimensions of the LSI reduction. Then, we merged together these preprocessed and quality‐controlled data from each day(batch) for downstream analysis.

For RNA‐seq data, we used FastQC (v. 0.12.1) to conduct quality control. Next, The Hisat2 aligner (v. 2.2.1) was utilized to align the FastQ files to Sus scrofa ensemble reference (v.11.1). After alignment, the SAM files were filtered and processed using Samtools (v.1.21). Finally, gene quantification was performed using HTSeq (v. 2.0.5), which counted the number of reads mapped to each gene in the Sus scrofa reference annotation (v.11.1.105).

### 2.5 Batch effect removal, pseudo‐bulk data generation, correlation and PCA analysis

For 10× multiome data, we observed batch effect present among the data from each day. To better mitigate the impact of batch effects in the data, we proposed a strategy integrating information from both scRNA‐seq and scATAC‐seq omics within each individual cell to perform batch effect removal. Firstly, we found integration anchors among the data from each day based on scRNA‐seq data which were projected into canonical correlation analysis space. Secondly, we integrated scRNA‐seq data from different days using these anchors. Thirdly, we transfered these anchors to integrate scATAC‐seq data from different days in supervised latent semantic indexing space, Then, we used ‘FindMultiModalNeighbors’ to calculate weighted shared nearest neighbours (WSNN) utilizing both scRNA‐seq and scATAC‐seq data after integration. Subsequent UMAP and clustering analyses were based on the results of the WSNN computation performed here.

For RNA‐seq data, we randomly divided the single‐cell transcriptome read count data from Day 0 of the 10× multiome into three groups and summed the counts within each group to obtain pseudo‐bulk gene expression data of cells in Day0. Then, we utilized ComBat function in sva (v.3.46.0) to remove the batch effect between these pseudo‐bulk data of cells in Day0 and the bulk RNA‐seq data of hepatocyte maintenance medium (HMM) and control. The Spearman correlation coefficients among these data were calculated by utilizing cor function in stats (v.4.2.2). Next, we project these data into principal component spaces using prcomp function in stats (v.4.2.2). To further mitigate the batch effect between pseudo‐bulk data and bulk RNA‐seq data, we performed dimensionality reduction visualization on the data using PC2 and PC3.

### 2.6 Clustering and cell type identification

Cell clustering was performed using the Louvain algorithm in Seurat (v.4.3.0) based on the WSNN graph. Cell types were identified based on cell‐type‐specific marker genes collected from references. Based on the expression profiles of marker genes, we merged clusters that appeared to represent the same cell type, resulting in 30 distinct cell clusters as shown in Figure 1D.

### 2.7 RNA velocity

First, data were preprocessed by filtering and normalizing with a minimum threshold of 10 counts per cell, 10 cells per count and logarithmic transformation. Second, based on spliced, unspliced and ambiguous RNA counts, the RNA velocity of each cell was calculated using velocyto (v.0.17.17) and scvelo (v.0.2.2) in ‘recover_dynamics’ mode. Third, RNA velocity ‘flows’ were reconstructed using parameters including the top 20 PCs, 50 nearest neighbours and UMAP visualization basis to study the differentiation process among these 30 cell clusters.

### 2.8 Gene set variation analysis

To explore the changes in pathway activity during hepatocyte reprogramming, we evaluated the activity of individual pathways along the cellular pseudotime using the normalized enrichment score method on Reactome pathways (https://reactome.org) through the GSVA R package (v1.34.0). The gsva function was supplied with a UMI matrix normalized using SCTransform. For the heatmaps in Figure 4B,D, we first collected gene sets in these representative pathways and then used the GSVA method to calculate the enrichment score. The colours in the heatmaps of Figure 4B,D indicate the enrichment level of each pathway.

### 2.9 TF analysis

We utilized raw counts as the input data and employed SCENIC (v.1.1.2–2) in R along with arboreto (v.0.1.5) in Python for the analysis of TF regulons. First, we converted gene symbols of Sus scrofa to gene symbols of Homo sapiens via orthologues. Second, specific database files and motif annotations for human were configured, including ‘hg19‐500bp‐upstream‐7species.mc9nr.feather’, ‘hg19‐tss‐centered‐10kb‐7species.mc9nr.feather’ and ‘motifAnnotations_hgnc_v9’ in RcisTarget (v.1.20.0). Third, the GRNBoost algorithm was employed to discern regulatory relationships between TFs and their target genes. Subsequently, SCENIC was utilized to construct co‐expression modules, form regulons and assess regulon activity on a per‐cell basis. The outcomes, encompassing gene set activity and regulon activity per cell, were visualized through heatmaps.

### 2.10 SCENIC+ analysis

In order to run SCENIC+ on the pig genome, a custom regions‐versus‐motifs database for pycistarget was built using aertslab scripts (github.com/aertslab/create_cisTarget_databases). The SCENIC+ clustered motif collection comprised of 10,249 PWM files was used (resources.aertslab.org/cistarget/motif_collections/v10nr_clust_public). The motif to TF annotation was adapted to pig by gene orthology using OrthoFinder^20^ following the method used in the study.^21^ The TF list referred to the one used in the study.^21^ The SCENIC+ pipeline was run following the tutorial (https://scenicplus.readthedocs.io/en/latest/tutorials.html). Briefly, we explored the linkage of predicted TF‐binding sites and enhancers to candidate target genes. Following the SCENIC+ pipeline, we created eGRNs with input from pycisTopic's imputed chromatin accessibility, pycisTarget's TF cistromes (i.e., a TF with its potential target regions) and the gene expression matrix. Linear correlation and gradient‐boosting machines were used to infer region–gene links (within a 150 kb space upstream and downstream of each gene) and TF–gene relationships. Then, we employed an enrichment analysis approach to assess whether the TF‐coexpression module significantly overlapped with genes recovered from motif/region‐based links and retained the optimal set of target genes and regions for each TF. A TF, along with its predicted target enhancers and regions, forms an enhancer regulon (eRegulon). Quantitative estimation of the driving force strength, based on the distance of TF expression along the pseudo‐time axis to its subsequent cellular state, was performed. Finally, we selected 28 positive eRegulons with TF‐cistrome correlation between −0.65 and 0.6 for downstream analysis and plotting. Especially, for the heatmap in Figure 3I, we first used SCENIC to build co‐expression gene modules and predict TF regulons. Then we scored the activity of these TF regulons by including gene set activity and the area under the curve. The colours in the heatmap indicate the activity levels of these TF regulons.

### 2.11 Total RNA isolation

The RNA extraction was performed using the Trizol reagent (Invitrogen) following the manufacturer's protocol. Briefly, each sample (1 × 10^7^ cells) was homogenized in 300 μL Trizol reagent. Two microlitre Linear polyacrylamide (Invitrogen) was added, then chloroform was added to the homogenized samples (0.2 mL of chloroform per 1 mL of Trizol). The tubes were vigorously shaken for 15 s and then incubated at room temperature for 2–3 min to allow phase separation. The samples were centrifuged at 12,000*g* for 15 min at 4°C and resulted in the separation of the mixture into a lower phenol‐chloroform phase, an interphase and an upper aqueous phase. RNA was in the aqueous phase. Then, the aqueous phase was transferred to a new tube. RNA was precipitated by adding 150 μL isopropyl alcohol and incubating at −20°C overnight. On the second day, the samples were centrifuged at 12,000*g* for 30 min at 4°C. The supernatant was discarded, leaving behind the RNA pellet. The RNA pellet was washed with 300 μL 75% ethanol. The samples were vortexed and centrifuged at 12,000*g* for 5 min at 4°C. The ethanol was discarded, and the RNA pellet was air‐dried for 5–10 min. The pellet was then dissolved in RNase‐free water by pipetting up and down gently. The concentration and purity of RNA were determined using a NanoDrop instrument.

### 2.12 cDNA synthesis and qRT‐PCR

For the cDNA synthesis, 2.0 μg of total RNA from each sample was used. The first‐strand cDNA was synthesized using the iScript cDNA Synthesis Kit (Bio‐Rad) according to its manuscript. The synthesized cDNA was either used immediately for qRT‐PCR or stored at −20°C for future use. The qRT–PCR reactions were set up using iQSYBR Green Supermix (Bio‐Rad). The primers are listed in Table S1. For data analysis, the cycle threshold (Ct) values, which represent the cycle number at which the fluorescence signal crosses the threshold, were determined for each reaction. Relative gene expression levels were calculated using the ΔΔCt method, normalizing against the expression of housekeeping genes.

### 2.13 Immunofluorescence staining

Cells were fixed using 4% neutral buffered formaldehyde. The fixation process involved incubating the cells at 4°C for 15 min. Post‐fixation, the cells were rinsed three times with phosphate‐buffered saline (PBS), each rinse lasting for 5 min. To reduce non‐specific binding, cells were blocked with 5% goat serum. This blocking step was carried out at 37°C in a humidified incubator for 30 min. After blocking, the serum was discarded. Cells were then incubated with the appropriately diluted primary antibody. This incubation was done overnight at 4°C to ensure optimal binding of the antibody to its specific antigen. Post‐primary antibody incubation, cells were washed three times with PBS, each wash lasting for 5 min, to remove any unbound antibodies. The cells were incubated with a diluted secondary antibody, specific to the primary antibody, for 1 h. This incubation step was performed at room temperature to allow for the binding of the secondary antibody to the primary antibody. Following the secondary antibody incubation, cells were stained with DAPI (4′,6‐diamidino‐2‐phenylindole) working solution for nuclear visualization. This staining was conducted for 10 min at room temperature, shielded from light. After DAPI staining, the cells were washed once with PBST (PBS with Tween) for 5 min and then three times with PBS, each wash lasting 5 min. The cells were then covered with an anti‐fade mounting medium to preserve fluorescence and prevent photobleaching. Finally, the stained cells were examined under a fluorescence microscope (ZEISS Axio Observer, Germany). Images were captured to analyse and document the fluorescence signals. We used the following primary antibodies: Albumin (BRTHYL; A100‐110A; 1:1000) and HNF‐4‐alpha (abcam; ab41898; 1:500).

### 2.14 Lipid staining

BODIPY 493/503 (4,4‐difluoro‐1,3,5,7,8‐pentamethyl‐4‐bora‐3a,4adiaza‐s‐indacene; Sigma) solution (1 mg/mL) was added to the HCM medium (1 mL per well of a 12‐well plate); 1 h later, the cells were washed with medium, and Hoechst was used to staining nucleus. Images were captured with fluorescence microscopy.

### 2.15 ALB ELISA of cell culture supernatants

The amount of ALB in cell culture supernatants was determined using an ELISA kit (Assaypro) in accordance with the manufacturer's instructions. Antibodies were purchased from BRTHYL (A100‐110A). Cells were cultured with a new medium for 24 h, and the supernatant was collected for analysis.

### 2.16 Hepatocyte transplantation

The transplantation of PPH into mice was conducted under the approval and guidelines of the Institutional Animal Care and Use Committee of Tsinghua University. The study utilized immune‐deficient, fumarylacetoacetate hydrolase (Fah)‐deficient NOD mice, which lacked B, T and natural killer cells due to disruption of Rag2 and Il2rg (FRGN mice), serving as recipients. FRGN mice were maintained on NTBC (Selleck) in their drinking water at a concentration of 10 mg/L. One day prior to transplantation, NTBC was withdrawn from the mice. To prepare the mice for hepatocyte transplantation, an adenovirus expressing urokinase plasminogen activator (Ad‐uPA, Yecuris) was administered via retroorbital injection. This was done 24 h before transplantation at a dosage of 5E7 plaque‐forming units per gram of body weight. The transplantation was performed via intrasplenic injection. Mice were anaesthetised with isoflurane, and a left‐flank incision was made to access the spleen. The hepatocytes, isolated immediately prior from pigs, were then injected into the spleen. After transplantation, NTBC was provided in the drinking water in cycles (7–10 days off NTBC, followed by 2–3 days on NTBC at a concentration of 8 mg/L). For surgical prophylaxis, amoxicillin (HeBei) was administered intraperitoneally immediately after transplantation and continued daily for 7 days. To accommodate the immune deficiency of the mice, Meloxicam (QILU ANIMAL HEALTH PRODUCTS) was provided for 7 days, followed by trimethoprim/sulfamethoxazole (TMP/SMX; Yecuris) in the drinking water continuously for another 7 days.

### 2.17 Immunohistochemistry

Liver tissues were fixed in a 4% paraformaldehyde solution for over 24 h at room temperature. This step ensures adequate preservation of tissue morphology and antigenicity. After fixation, tissues were dehydrated through a graded alcohol series, cleared in xylene and embedded in paraffin. Tissue blocks were sectioned at 5‐μm thickness using a microtome, providing thin slices for detailed microscopic examination. The tissue sections were deparaffinized in xylene and rehydrated through a graded alcohol series to water. This step removes the paraffin and gradually brings the tissues to an aqueous state, essential for antigen retrieval and staining. Antigen retrieval was performed by heating the sections in citrate buffer (pH 6.0). This process unmasked the epitopes by breaking the cross‐links formed during the fixation, enhancing antibody accessibility. To reduce non‐specific background staining, sections were incubated with a blocking solution, typically 5% goat serum, for 30 min at room temperature. Sections were incubated with the primary antibody, diluted as per the manufacturer's recommendations, overnight at 4°C. This allowed for the binding of the antibody to specific antigens in the tissue. After primary antibody incubation, sections were washed with PBS to remove any unbound antibodies. A biotinylated secondary antibody, specific to the primary antibody, was applied to the sections for 1 h at room temperature. This antibody was conjugated to an enzyme like horseradish peroxidase or alkaline phosphatase. The bound secondary antibody was visualized using a chromogenic substrate (DAB for peroxidase), resulting in a colorimetric reaction at the site of antigen–antibody binding. Sections were counterstained with haematoxylin to visualize the nuclei, providing a contrast to the specific staining. After a final washing step, the slides were mounted with a coverslip using a mounting medium. For imaging and analysis, stained tissue sections were examined under a microscope (e.g., ZEISS Axio Observer, Germany). Images were captured, focusing on areas of interest. For quantitative analysis, the liver reconstruction ratios were quantified using image analysis software (ImageJ). The area of positive staining was measured and compared to the total tissue area to assess the extent of liver regeneration or specific protein expression.

### 2.18 Tissue immunostaining

We dewaxed paraffin‐embedded sections in xylene (5 μm‐thick) and subjected to sodium citrate (10 mM, PH = 6.0) at 100°C for 15 min. After that, sections were blocked in 0.3% Triton X‐100 diluted with PBS for 15 min. Then, sections were blocked with 3% bovine albumin for 1 h. We incubated primary antibodies overnight at 4°C in 3% bovine albumin. The next day, sections were first washed three times with PBST and incubated with fluorescent‐conjugated secondary antibodies diluted in 3% bovine albumin for 1 h at room temperature. Finally, we utilized 5 μg/mL DAPI for nuclear staining. We used the following primary antibodies: Albumin (BRTHYL; A100‐110A; 1:1000), HNF‐4‐alpha (Abcam; ab41898; 1:200), FAH Polyclonal (Invitrogen; PA5‐42049; 1:400). They were incubated overnight at 4°C in 3% bovine albumin.

### 2.19 ELISA of porcine serum albumin

Porcine serum albumin levels were determined with the ELISA Quantitation Set (catalogue no. E80‐129; Bethyl). Blood (3 mL) drawn by tail clipping was immediately diluted 1:100 in sample diluent; HSA concentration was determined by ELISA with a Pig‐specific ALB antibody.

### 2.20 Survival study

The study utilized 8–12 week‐old FRGN mice (both male and female, equally distributed between groups). These mice are characterized by a deficiency in the Fah gene and lack B, T and natural killer cells due to disruption of Rag2 and Il2rg.

Mice were initially maintained on NTBC (nitisinone) in their drinking water at a concentration of 10 mg/L. One day before hepatocyte transplantation, NTBC was withdrawn. Additionally, mice received an adenovirus expressing urokinase plasminogen activator (Ad‐uPA; dose: 5E7 plaque‐forming units per gram body weight) via retroorbital injection to induce liver damage and enhance engraftment. Mice underwent hepatocyte transplantation as described in the hepatocyte transplantation section. 7 days after transplantation, mice were subjected to cycles of NTBC administration: 7–10 days without NTBC followed by 3 days with NTBC at a dose of 8 mg/L. This cycling aimed to support the survival of transplanted hepatocytes and the regeneration of the liver. Mice were monitored daily for general health and survival. Survival data were recorded from the day of transplantation until a predetermined endpoint or natural death.

Survival data were plotted as a Kaplan–Meier survival curve. The *x*‐axis represented the time (days) post‐transplantation, and the *y*‐axis represented the percentage of surviving mice.

### 2.21 Statistical analysis

Initial analysis involved calculating descriptive statistics, such as mean and standard deviation or standard error of the mean, for each dataset. This provided an overview of the data distribution and variability. For comparing data between two groups, Student's *t*‐test was used. The *t*‐test was chosen for datasets that passed the normality test. An unpaired, two‐tailed format of the *t*‐test was utilized. Survival data were analysed using the Mantel–Cox log‐rank test. This test compared the survival distributions of two groups and was particularly useful for the analysis presented in the survival curve section of the study. A *p*‐value of less than 0.05 was considered statistically significant. This threshold was applied to all statistical tests to determine the significance of the findings. Statistical analyses were conducted using appropriate statistical software (GraphPad Prism).

## 3 RESULTS

### 3.1 A high‐quality scRNA + scATAC library was prepared using 10× multiome technology

Primary hepatocytes tend to undergo dedifferentiation after isolation from the liver and subsequent culture in vitro with HCM. Morphologically, the primary hepatocyte lost its typical polygonal shape and distinct nuclear features (Figure 1A). A series of hepatic essential genes were rapidly downregulated, while AFP, a dedifferentiation marker, was upregulated, indicating dedifferentiation (Figure S1A). Protein levels of ALB and HNF4α also decreased by Days 3 and 5, with only slight re‐expression by Day 7 (Figure S1B). Lipid synthesis significantly declined (Figure S1C), further supporting the dedifferentiation of hepatocytes during this process. To comprehensively understand the dynamic changes during hepatocyte dedifferentiation in vitro, we utilized 10× multiome technology (Figure 1B) to analyse RNA transcription and chromatin accessibility at the single‐cell level. Following the manufacturer's protocol, we optimized the extraction of cell nuclei and identified 0.2% NP40 as the optimal condition to effectively balance cell penetration and nuclear preservation. Following this step, 10× multiome was employed for library preparation and sequencing. We obtained 49,091 cells, with a median gene count per cell of 2872 for RNA data and an average transcription start sites enrichment score of 16 for ATAC data (Figure S2A). After quality control, we retained 39,803 single cells for downstream analysis, including 11,977, 6492, 7535, 7676 and 6123 cells from the D0, D1, D3, D5 and D7 time points, respectively.

### 3.2 The cell population identification during in vitro culture

To study the cell heterogeneity during the hepatocyte dedifferentiation process, we used Seurat^22^ and UMAP^23^, ^24^ for cell population identification. 10× multiome provided both RNA and ATAC data for the same cell, contributing to more accurate cell‐population identification than scRNA‐seq or scATAC‐seq alone.^16^ As shown in Figure 1C, 10× multiome identified small clusters missed by RNA‐seq alone, which were named cluster 27 and cluster 28 (Figure 1D). Next, we analysed different expression genes and pathway enrichment over different days and found that hepatic genes and liver function‐related pathways (e.g., glycogen, lipid, amino acid metabolism) gradually declined during in vitro culture, once again confirming the occurrence of dedifferentiation (Figure 1E). Notably, four out of the 16 pathways (specifically those related to lipid metabolism) (Figure 1E) and several hepatic genes (Figure S2B) exhibited signs of recovery in D7 cells. This observation suggests that D7 cells may partially regain hepatic functions and provides valuable insight into potential strategies for maintaining hepatocyte functionality. Overall, we clustered cells into 30 populations based on joint RNA and ATAC data (Figure 1D). Next, we analysed the changes within these clusters to explore cellular dynamics during in vitro culture.

### 3.3 Cluster distribution during the hepatocyte‐dedifferentiation process

**FIGURE 2:**
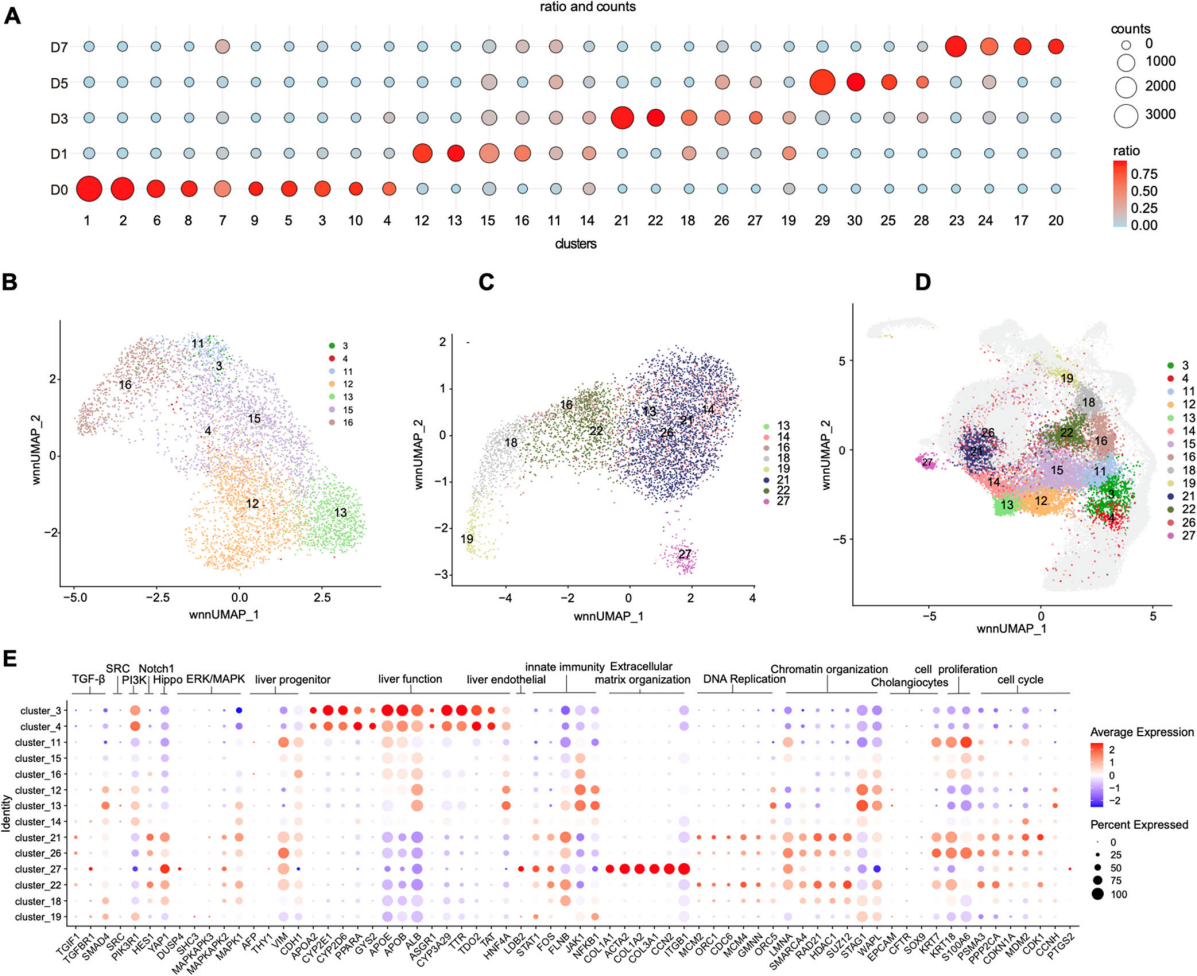
The key molecular changes of the early dedifferentiation stage. (A) The dot plot illustrates the proportion of cells sampled on different days within each cell cluster, with larger bubbles indicating a greater number of cells and a redder colour indicating higher proportions. (B) Uniform manifold approximation and projection (UMAP) plot showing the cell cluster of D1 dominate. (C) UMAP plot showing the cell cluster of D3 dominate. (D) Position D1 and D3 dominate clusters in the UMAP of total cells. (E) The dot plot of representative marker gene expression levels in D1 and D3 clusters. The size of the dot indicates the percentage of cells in each population expressing each gene. The colour of the dot indicates the gene expression levels. A larger size indicates a higher proportion of expressing cells, and a redder colour signifies a higher average gene expression level.

To explore the distribution of these 30 clusters during dedifferentiation, we first analysed their composition across different cultivation days (Figure 2A, Table S1). The cells of D0 were all primary liver cells, which typically exhibited highly characteristic gene expressions (Figure S3A).^25^, ^26^, ^27^, ^28^, ^29^ These cells comprised hepatocytes expressing *ALB* and *CYP3A29* ^30^ (clusters 1, 2, 4 and 5), cholangiocytes marked by *CFTR*, *CD24* and *SOX9* (cluster 7), Kupffer cells characterized by *C1QA*, *C1QB* and *C1QC* (cluster 8),^31^ and fibrotic cells identified by *COL1A2* and *ACTA* (cluster 9).^32^ After cultured in vitro, clusters 11, 13, 15, 16, 18, 22, 26 and 27, predominantly from D1 and D3, lost their marker gene expressions, and the correlation between cell composition and culture days also became blurred. With the extension of culture time, for clusters 23, 24, 17, 20, 29, 25 and 28 that predominantly observed on D5 and D7, the correlation between cell composition and culture days became clear again. This dynamic distribution highlighted significant heterogeneity in both the timing and the molecular pathways governing cell differentiation.

### 3.4 The molecular characteristics of cell identity loss in the early stage

To understand this process in‐depth, firstly, we analysed the molecular changes at the early stage. We respectively displayed the cell clusters dominated by D1 or D3 and their positions in the UMAP of total cells (Figure 2B–D). Initially, we used Monocle2^33^ to construct the cell trajectory among cells from D0 to D7 as shown in Figure S3B. However, the cell clustering displayed by Monocle2 is quite dense, making it difficult to clearly present each cell cluster to the readers. Then we used scVelo to analyse the direction of cell differentiation among cell clusters as shown in Figure S3C. These analyses indicated that clusters 3, 4 and 11 are the transitional state clusters between D0 and D1 (Figure 2A,D), with gene expression changes characterized by a reduction in liver function‐related genes and activation of cell proliferation‐related genes (Figure 2E). Notably, cluster 11 uniformly contained cells from each day (Figure 2A), further suggesting the transitional nature of this group of cells. Clusters 12, 13, 15 and 16 were adjacent to cluster 11 (Figure 2B,D). Among them, cluster 16 was characterized by genes related to innate immunity, chromatin organization and the initiation of DNA replication (Figure 2E). In the other direction, cluster 15, along with clusters 12 and 13 (Figure 2B,D), showed significant activation of genes involved in the JAK–STAT, NFkB and TGF‐β signalling pathways, highlighted by genes such as *JAK1*, *NFKB1* and *SMAD4* (Figure 2E).^34^ There was also a noticeable rise in chromatin structural genes such as *SMARCA4, STAG1* and *WAPL* ^35^, ^36^, ^37^ in these cell clusters, indicating substantial chromatin reorganization along this trajectory (Figure 2E).

In the clusters of D3, the differences among various cell clusters further increased. Adjacent to cluster 13, in cell clusters such as 14, 21 and 26 (Figure 2C,D), the expression of inflammatory genes gradually subsided, while genes related to DNA replication (*GMNN*, *MCM* family and *ORC* family^38^), cell cycle (*CCNH* and *CDK1*),^39^ and chromatin restructuring (*SMARCA4, RAD21, HDAC* and *SUZ12*)^35^, ^40^ became active (Figure 2E). Next to cluster 16, particularly clusters 18 and 19 (Figure 2C,D), genes related to DNA replication, cell cycle and chromatin restructuring transiently increased and then decreased, while genes associated with innate immunity, chromatin organization and other related processes gradually strengthened (Figure 2E).

From the above analysis, it was evident that the majority cell population at D3 was characterized by active cell proliferation. At the same time, due to the progressing dedifferentiation, the differences among them also gradually increased. Among them, the most unique group, cluster 27 (Figure 2C,D), a distinct cell cluster revealed by scATAC‐seq, exhibited a fibroblast‐like state with high expression of extracellular matrix genes (e.g., *PDGFRB, COL1A1, ACTA2, COL1A2, COL3A1, COL6A1, CCN2*) (Figure 2E, Figure S4A) and a significant overlap with the regulons of cluster9 from D0 (a mixture cluster representing fibrotic cells and LSEC) (Figures S3A and S4B and Table S2).

### 3.5 The molecular characteristics of dedifferentiation plasticity in late stage

**FIGURE 3:**
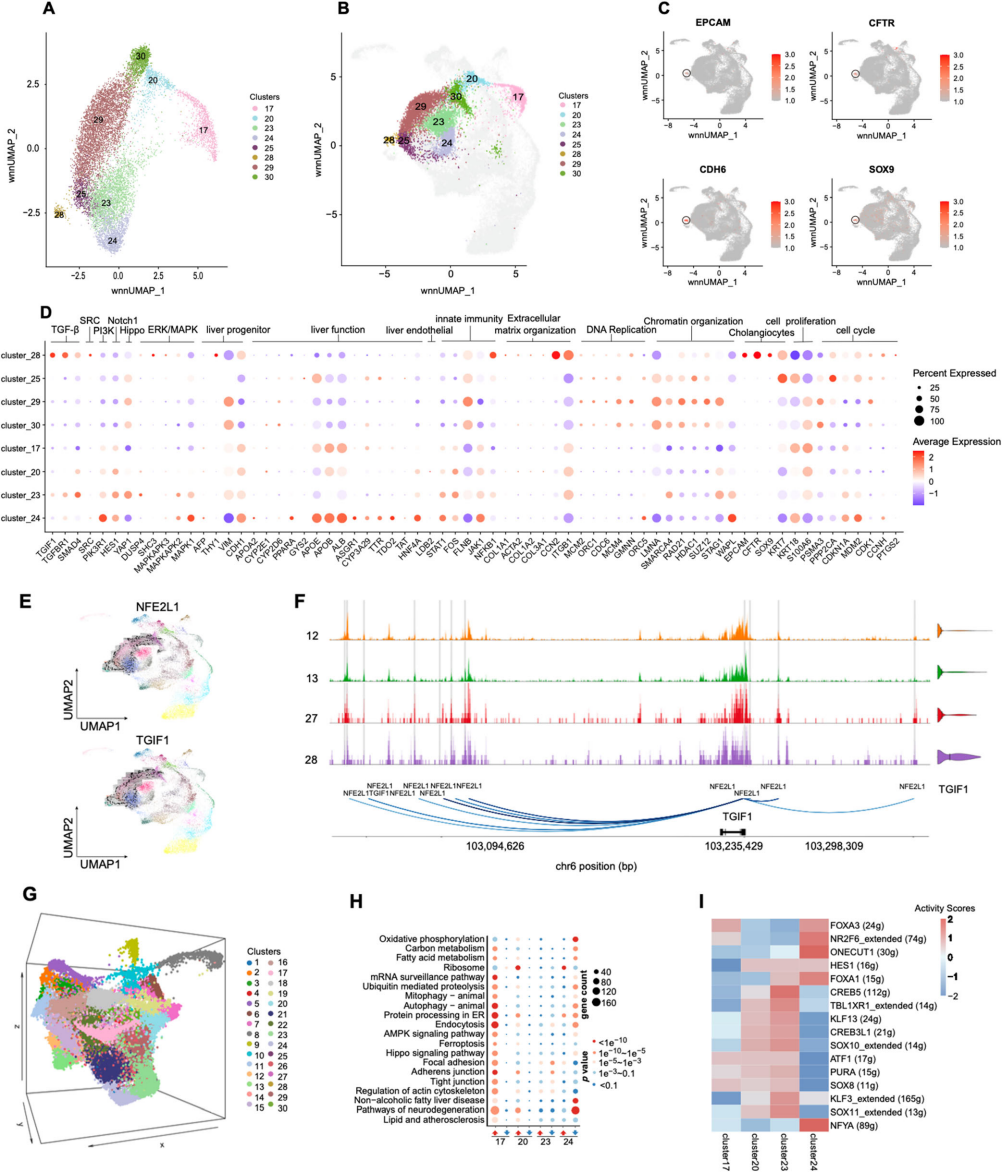
The molecular characteristics of plasticity in late stage. (A) Uniform manifold approximation and projection (UMAP) plot showing the cell cluster of D5 and D7 dominate. (B) Position D5 and D7 dominate clusters in the UMAP of total cells. (C) UMAP shows the expression distribution of clolangiocyte‐related genes that reappear on Cluster 28. (D) The dot plot of representative marker gene expression levels in the D5 and D7 cluster. The size of the dot indicates the percentage of cells in each population expressing each gene. The colour of the dot indicates the gene expression levels. A larger size indicates a higher proportion of expressing cells, and a redder colour signifies a higher average gene expression level. (E) Differentiation force of transcription factors (TFs) on TGIF1 gene predicted by SCENIC+. The SCENIC+ develops a method to quantify the differentiation force of TFs by ordering cells along a pseudotime axis and measuring the distance to their future cell based on current TF expression and target gene expression. (F) Chromatin‐accessibility profiles across cell clusters on TGIF1 gene. The RNA expression of TGIF1 were listed on the right side of each cell cluster. Blue arcs connect putative regulatory regions to the transcription start sites of regulated genes. TFs predicted to bind these regions are annotated at the arc endpoints. Region‐gene gradient‐boosting machine feature importance scores are encoded as colours (from light to dark blue). (G) 3D UMAP Visualization of cell clusters. (H) The dot plot illustrating the differential gene enrichment in KEGG pathways among the four primary clusters in Day7. Larger dots signify a higher number of enriched genes, with increasing red colour indicating greater significance. On the *x*‐axis, upward arrows adjacent to cluster labels denote the enrichment of upregulated gene sets, while downward arrows indicate the enrichment of downregulated gene sets. (I) The heatmap of representative regulons' activity scores of clusters 17, 20, 23 and 24 in D7.

During D5–D7 the hepatocytes cultured ex vivo continued to change. Clusters 25, 28, 29 and 30 were primarily distributed on D5 (Figures 2A and 3A,B). Among these, clusters 25 demonstrated vigorous cell proliferation, as evidenced by the high expression of genes related to DNA replication (*GMNN*, *MCM* family and *ORC* family) and cell cycle regulation (*CDC6* and *CDK1*) (Figure 3D).^39^ In contrast, clusters 29 and 30 showed decreased cell cycle regulation‐related genes while increased *YAP, VIM, FLNB* and chromatin reorganization‐related genes, indicating that the cells were prepared for state remodelling. Unexpectedly, cluster 28, another distinct cell cluster revealed by scATAC‐seq, exhibited distinct characteristics of cholangiocytes, marked by the expression of specific markers such as *EPCAM*, *KRT7*, *CFTR*, *CD24*, *SOX9* and *KRT8* (Figure 3C,D). Given the distinct features of cluster 28, we further utilized SCENIC+ to integrate information in scRNA‐seq and scATAC‐seq and predicted the candidate TFs and enhancers (Figure S5). The analysis predicted that the driving TFs in cluster 28 were *KLF5, ELK3, NFE2L1*, etc. Specifically, for *TGIF1*, a representative gene of the TGF‐β signalling pathway essential for the development of cholangiocytes^41^ was analysed, where NFE2L1 was predicted to have dense binding in the regulatory region of *TGIF1* (Figure 3E,F). These results indicated that trans‐differentiation may occur during hepatic dedifferentiation.

At D7 of ex vivo culture, clusters 17, 20, 23 and 24 appeared widely dispersed on the 2D and 3D UMAP plots (Figure 3A,G and Figure S6). Although all of these clusters demonstrated increased activity of the Notch pathway (Figure 3D), they displayed notable differences in other critical pathways (Figure 3H) and TFs (Figure 3I).^42^ Regarding gene expression, cluster 17 primarily expressed *KRT18* and *S100A6* (Figure 3D) and maintained a certain level of cell proliferative capacity. Various signalling pathways in cluster 23 were broadly activated (Figure 3D). Intriguingly, cluster 24 showed an upregulation of genes linked to hepatic functions, such as *ALB*, *APOE*, *APOB* and *HNF4A* (Figure 3D and Figure S2B). Additionally, the Hippo signalling pathway, which was associated with hepatic dedifferentiation, was repressed. As the Hippo signalling pathway can be regulated by cell density,^43^ the high cell density of D7 cells (Figure 1A) may repress the Hippo signalling pathway (Figure 3H). However, although cluster 24 exhibited certain hepatic function‐related gene characteristics, there was a long distance between cluster 24 and mature hepatocytes in D0 on the 3D UMAP (Figure 3G and Figure S6), indicating a significant difference from mature hepatocytes.

### 3.6 Cell–cell communication during hepatocyte dedifferentiation

To further understand the mechanisms during dedifferentiation, we predicted the ligand‐receptor interactions between the cell types using the CellChat^44^ and its internal databases. This approach revealed the most interactions among these 30 clusters. Pivotal interactions among clusters in the D1–D3–D5–D7 were illustrated by dot plots respectively in Figure S6. Among them, ANGPTL4‐dependent signalling was present among all D1 clusters, and ANGPTL4–SDC2 interactions were particularly prominent (Figure S7). As ANGPTL4 secreted by MSCs inhibited macrophage proinflammation,^45^ we inferred that it also had anti‐inflammatory effect during dedifferentiation. As mentioned earlier, we have found that inflammation‐related genes were upregulated in clusters 12 and 13 (Figure 2E). These results suggested that both inflammation and anti‐inflammation might regulate cell state transition coordinately. Enriched Ligand‐Receptor pair obtained in the D1 cluster also included myelin protein zero‐like 1 (MPZL1) + MPZL1. MPZL1 was a transmembrane glycoprotein that promoted migration of hepatocellular carcinoma cells and was involved in extracellular matrix‐induced signal transduction.^46^, ^47^ MPZL1 was reported to induce Src kinase‐mediated phosphorylation and activation of the pro‐metastatic protein, cortactin, promoting HCC cell migration.^46^, ^47^ Cluster 27, as a group of cells with LSEC characteristics, exhibited the hepatocyte growth factor/c‐MET combination. When c‐MET bound to its ligand hepatocyte growth factor, tyrosine residues in the cytoplasm can undergo autophosphorylation, thereby activating tyrosine kinases.^48^ Subsequently, various effector proteins in the cytoplasm were rapidly phosphorylated, activating multiple intracellular signalling pathways such as PI3K‐Akt, Ras‐MAPK and STAT3 pathways,^49^ which was also activated in this cluster.

### 3.7 Cross‐species comparison of regulators during hepatocyte dedifferentiation

**FIGURE 4:**
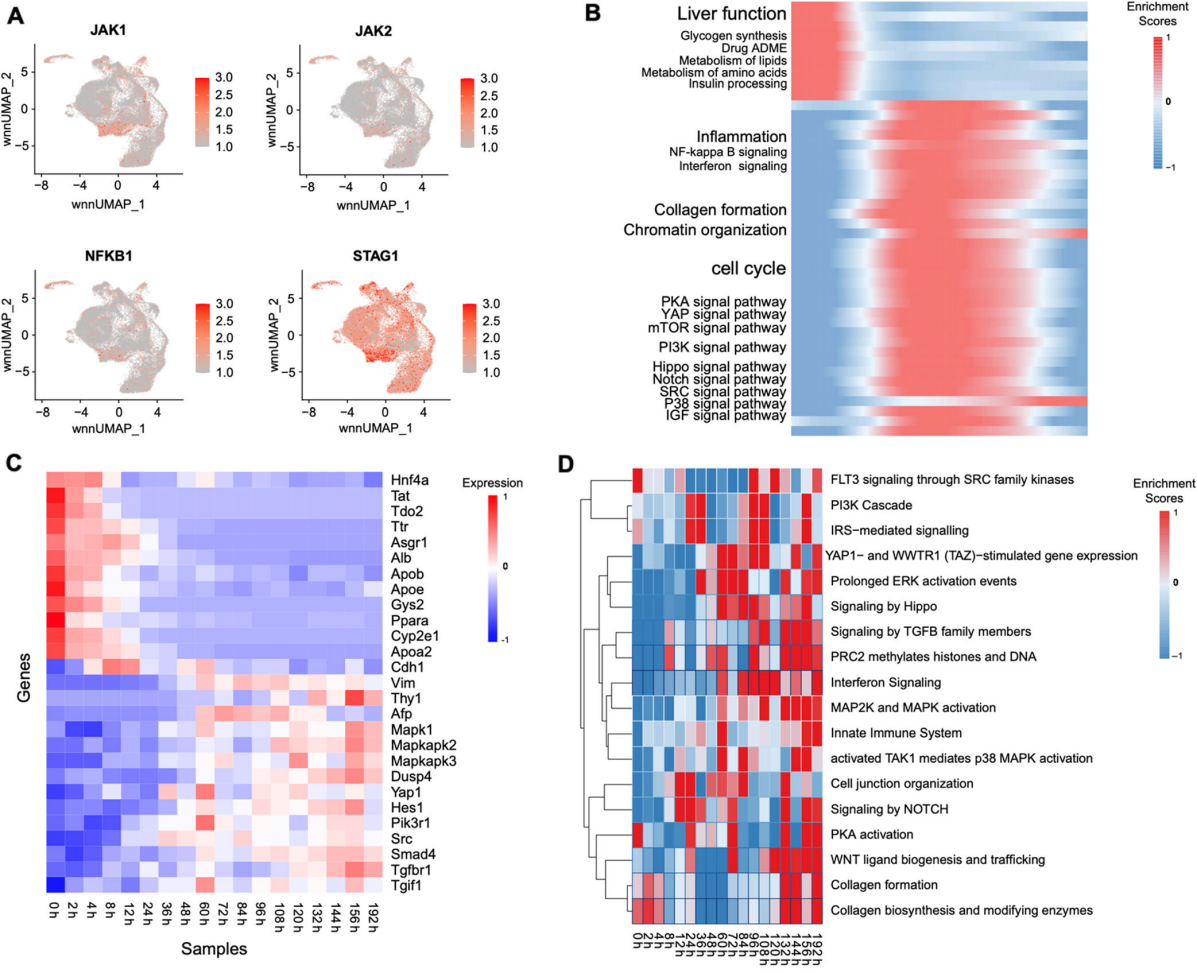
Cross‐species comparison of signalling pathway provides intervention targets. (A) Uniform manifold approximation and projection (UMAP) distribution of some genes indicates a drastic change in the state of D1 cells. (B) The heatmap represents the activation levels of representative pathways in each cell along the cell in D1. (C) The heatmap of representative marker gene expression levels in mouse hepatocytes cultured in vitro 0 h–192 h. (D) The heatmap represents the activation levels of representative pathways in mouse hepatocytes cultured in vitro 0 h–192 h.

Through the aforementioned analysis, we observed that porcine hepatocytes began to proliferate and lost hepatic functions once cultured in vitro. After reaching full confluency, some cells could partially re‐differentiate. Since porcine hepatocytes initiated the loss of functional characteristics and commenced proliferation as early as D1 of in vitro culture, it was imperative to meticulously characterize the cellular state changes on D1 to identify suitable pathways and genes for intervention. Our pathway analysis based on single cells from D1 revealed significant activation of several pathways, including PKA, mTOR, PI3K, Hippo,^43^ Notch,^50^ Src,^51^ P38 and IGF^10^ signalling pathways (Figure 4A,B). As numerous of them were also reported in other species, it indicated the accuracy of our bioinformatic analysis and the conservation regulations among different species.

To completely assess the conservation of these pathway changes across species, we further compared the dedifferentiation data of porcine hepatocytes with that of murine hepatocytes. This comparison revealed that the relevant genes and pathways were also upregulated during the 192‐h in vitro culture of murine hepatocytes (Figure 4C,D). Notably, during the dedifferentiation process of murine hepatocytes, there was a sustained high expression of inflammation and collagen‐related genes, while liver function‐related genes consistently decreased (Figure 4C), indicating an inability to regain hepatic characteristics. This contrasted with that of the porcine hepatocyte, highlighting species‐specific differences in the dedifferentiation process of porcine hepatocytes.

Besides, these findings indicated the necessity of targeted interventions at the early stages of hepatocyte culture to prevent dedifferentiation and promote the maintenance of hepatic functions.

### 3.8 Potential target validation with chemical compounds

**FIGURE 5:**
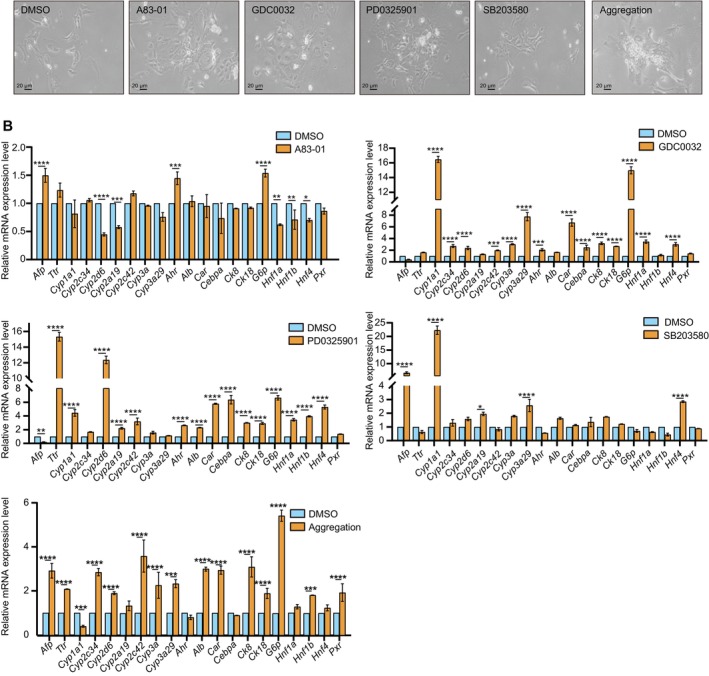
Potential target validation with chemical compound. (A) Morphologies of primary hepatocytes treated with compounds of A83‐01, GDC0032, PD0325901, SB203580 and the condition of aggregation for 5 days. The control sample was treated with DMSO. (B) The mRNA expression level of hepatic genes in primary hepatocytes treated with the compound of A83‐01, GDC0032, PD0325901, SB203580 and the condition of aggregation for 5 days. The control sample was treated with DMSO. *p ≤ 0.05, **p ≤ 0.01, ***p ≤ 0.001 and ****p ≤ 0.0001.

Based on the above analysis, we focused on the highly enriched target pathways that may influence hepatocyte dedifferentiation (Figure S8) and used small molecules to suppress the dedifferentiation of porcine hepatocytes. It was shown in Figure 5A that hepatocytes treated with the inhibitors of PI3K pathway (GD0032), ERK pathway (PD0325901), Src (SB203580) and TGF‐beta pathway (A83‐01) displayed a much better morphology compared with that of the control group. Specifically, cells in those conditions exhibited a smoother boundary, and most of the cells had clear nuclei. Besides, those cells showed a little cubic just as primary hepatocytes cultured at the D1 (Figures 1B and 5A). Additionally, single‐cell analysis showed partial hepatic gene upregulation in certain cell clusters of Day 7 (Figure 3D). Immunostaining and BODIPY staining also revealed that cells with high HNF4α expression and lipid droplets, which are the key characters of hepatocytes, were in dense areas (Figure S1B,C), suggesting that cell aggregation may enhance hepatic function. In this case, clustered hepatocytes were seeded and tested. As expected, aggregated cells were also morphologically better than those of the control group.

To explore the impact of those treatments on the mRNA expression of hepatocyte‐related genes, we examined the mRNA expression of *CYP450* genes. As shown in Figure 5B, treatment with A83‐01 specifically increased the expression of *CYP2C42*, whereas PD0325901 treatment broadly upregulated several *CYP450* genes including *CYP1A1*, *CYP22D6*, *CYP2A19* and *CYP2C42*. Similarly, the GDC0032 treatment significantly enhanced the expression of a wide array of *CYP450* genes, notably *CYP1A1*, *CYP2C34*, *CYP3A*, *CYP3A29*, *CYP2D6* and *CYP2C42*. In contrast, SB0325901 treatment specifically elevated the expression levels of *CYP1A1*, *CYP2A19* and *CYP3A29*. Furthermore, the treatment of aggregation led to an upregulation in *CYP2C34*, *CYP3A*, *CYP3A29*, *CYP2D6* and *CYP2C42* (Figure 5B).

Furthermore, we explored the expression of nuclear receptors such as *CAR*, *AHR* and *PXR*, known as upstream regulators of CYP450 genes. Consistent with the above results, our findings revealed that PD0325901 and GDC0032 treatments up‐regulated the expression of *AHR* and *CAR*, while A83‐01 treatment only increased the expression of *AHR*. Interestingly, SB203580 showed no effect on the expression of the tested nuclear hormone receptors, suggesting an alternative regulatory mechanism for CYP450 genes. Notably, cell clusters were found to significantly increase the expression of *PXR* and *CAR* (Figure 5B).

For hepatic genes, PD0325901 stood out by significantly elevating the expression of the hepatocyte‐specific marker gene *ALB*, while simultaneously diminishing *AFP* expression, suggesting its maintenance effect. Furthermore, PD0325901 and GDC0032 exhibited a shared propensity to enhance the expression of a spectrum of hepatic markers, such as *CK8*, *CK18*, *HNF4*, *HNF1α* and *HNF‐1β*. SB203580 primarily augmented *HNF4* expression without significant alteration of other markers. Additionally, treatments involving cell clusters led to a pronounced up‐regulation in the expression of hepatocyte markers *ALB*, *CK8*, *CK18* and *HNF1β*, indicative of a promotion in hepatocyte differentiation. Notably, all treatments, barring SB203580, significantly elevated the transcription levels of *G6P* genes, thereby underscoring their substantial role in modulating hepatic glucose metabolism (Figure 5B).

In sum, based on the 10× multiome data, we identified several relevant targets and validated them with small molecules on porcine hepatocyte maintenance.

### 3.9 Enhanced maintenance of primary hepatocyte through chemical combination

**FIGURE 6:**
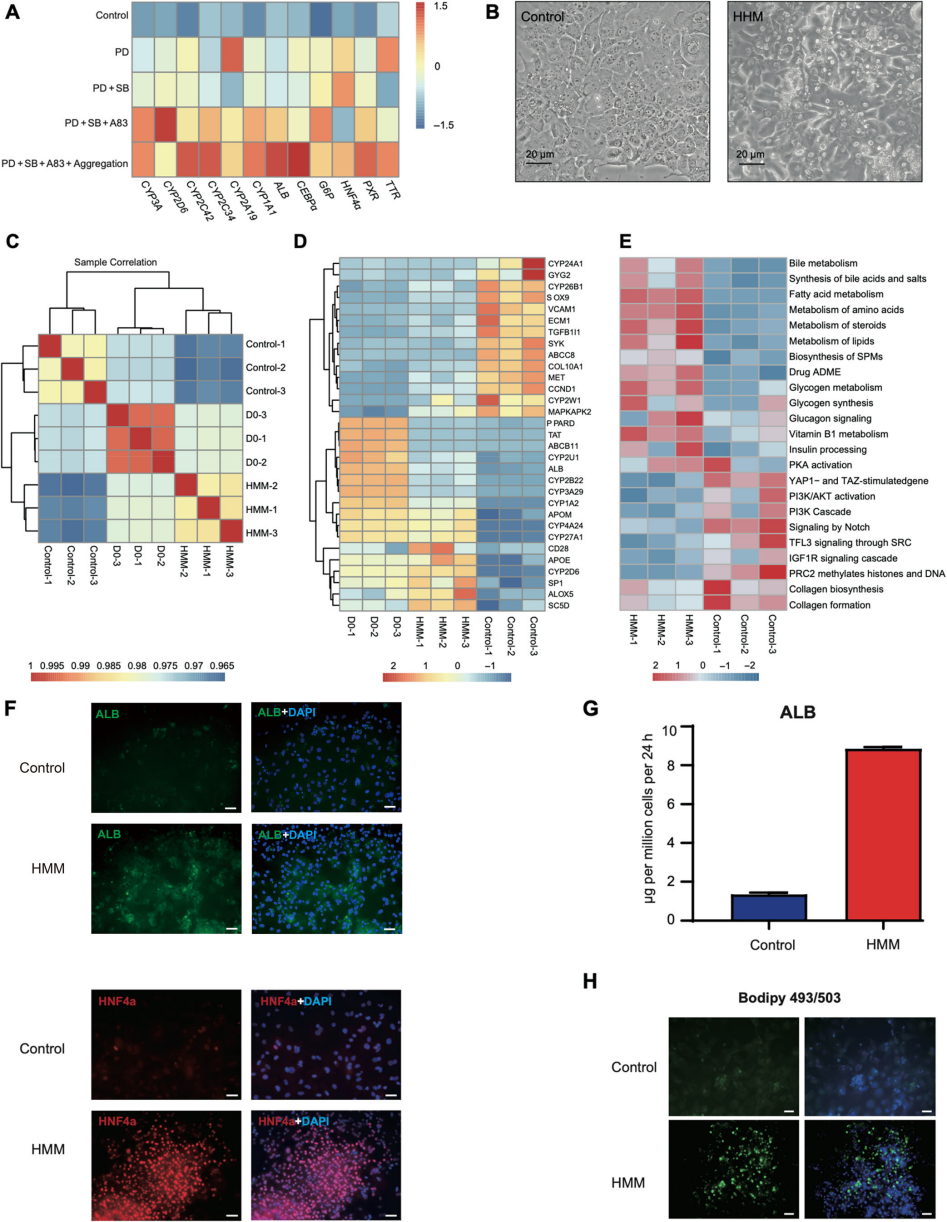
Hepatocyte maintenance medium (HMM) optimization and efficacy assessment. (A) Heatmap shows the expression of hepatic genes in the hepatocyte cultured with different chemical combinations. PD indicates PD0325901, SB indicates SB203580 and A83 indicates A83‐01. The expressions were measured with RT‐PCR, and all experiments were repeated with three samples for three times. (B) Morphologies of primary hepatocytes, which were treated with Control and HMM for 5 days. The control sample was treated with DMSO. (C) Correlation matrix illustrating the relationships among D0, HMM and Control samples. (D) The expression profile of key hepatic genes and dedifferentiation‐related genes in D0, HMM and Control samples. (E) The heatmap represents the activation levels of representative pathways in HMM‐treated and the control samples. (F) The Immunostaining of ALB and HNF4α in hepatocytes cultured with control medium and HMM medium at Day 5. Scale bars, 50 μm. (G) ELISA shows the ALB secretion ability of hepatocytes cultured with control medium and HMM medium at Day 5. (H) The BODIPY 493/503 staining of hepatocyte cultured with control medium and HMM medium at Day 5. Scale bars, 50 μm.

The limited efficacy of single compounds in maintaining hepatocytes led us to explore a multi‐target approach using a combination of small molecules to further improve the effect of hepatocyte maintenance. Initially, the effects of validated chemical compounds were assessed, and PD0325901 was selected as the base. Building on this, we conducted further screening. After three rounds of optimization, we developed a combination of three compounds. To further improve the culture conditions, cell aggregation was included, and it significantly enhanced the outcomes (Figure 6A). Ultimately, we discovered a specific combination (PD0325901, A83‐01, SB203580 and aggregation) that could significantly inhibit PPH dedifferentiation in the cell‐aggregation context, which we named HMM. Morphological analysis (Figure 6B) showed that hepatocytes treated with this condition retained characteristic features of hepatocyte (e.g., clear nuclei and cuboid shape), indicative of a quiescent state. In contrast, single cells treated with DMSO rapidly lost hepatic morphology (Figure 6B).

To molecularly understand the effect of this condition, we initially analyse the transcriptomes of cells cultured with HMM and control medium. It was found that HMM‐treated cells exhibited a higher correlation with Day 0 samples compared to that of control cells (Figure 6C). Additionally, key hepatic genes, such as *ALB* and *CYP2D6*, were highly expressed in HMM‐treated cells compared to the control group (Figure 6D). In contrast, genes related to the cell cycle (*CCND1*), fibrosis (*COL10A1*) and dedifferentiation (e.g., *TGFB1*, *MAPKAPK2*) were downregulated in the HMM‐treated samples compared to controls. Pathway analysis further confirmed upregulation of crucial hepatic functions, such as lipid metabolism and drug metabolism, in HMM‐treated cells compared to that of the control (Figure 6E). At the protein level, hepatic genes such as ALB and HNF4α were significantly upregulated compared to the control samples (Figure 6F). Moreover, key hepatic functions, including ALB secretion and lipid synthesis, were markedly improved in cells cultured with HMM compared to those in the control medium (Figure 6G,H). This finding suggests that the HMM condition inhibits hepatic dedifferentiation effectively and that the cells cultured with HMM are much closer to primary hepatocytes, comparing with that of the control group.

**FIGURE 7:**
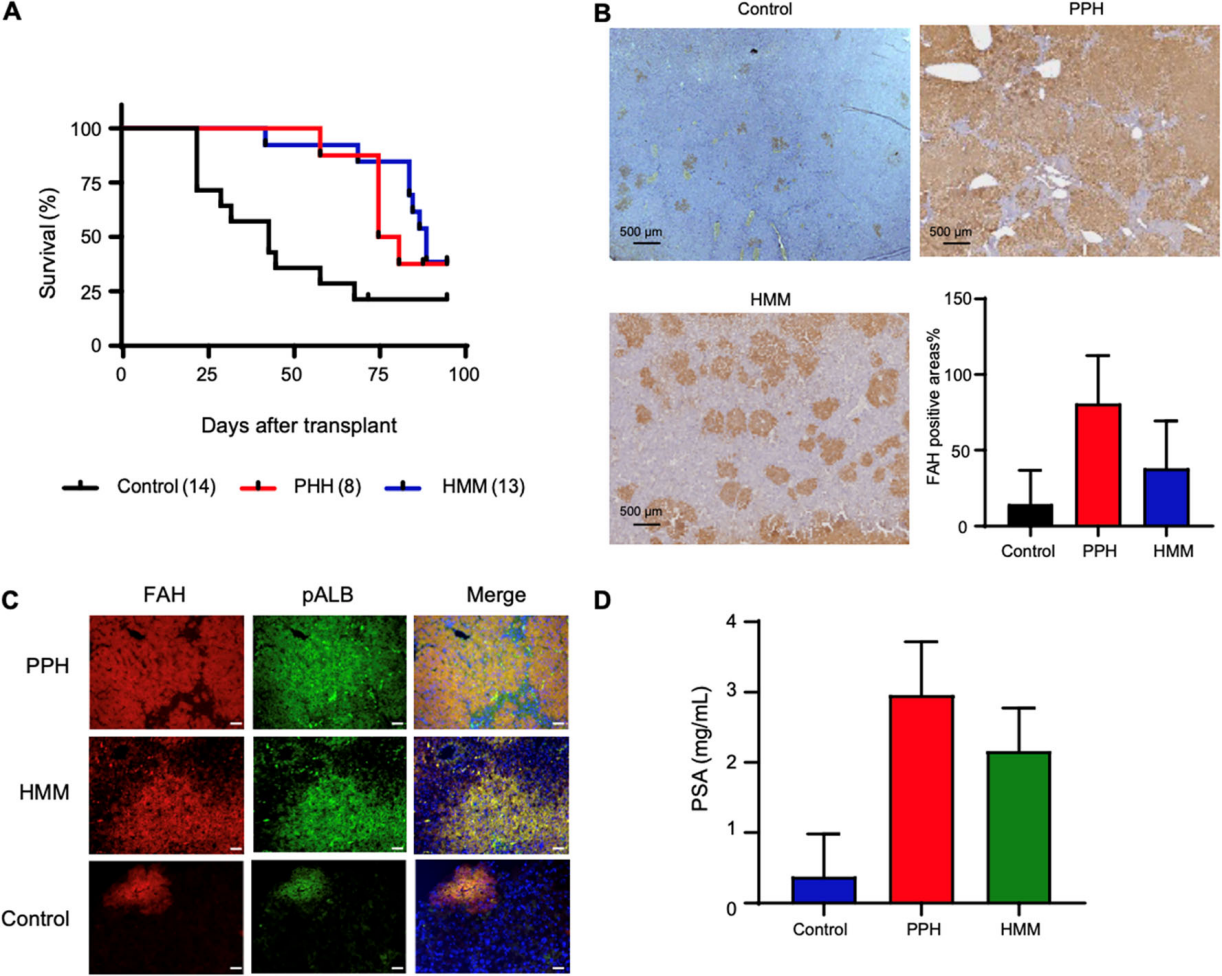
Enhanced maintenance of primary hepatocyte through chemical combination. (A) Kaplan–Meier survival curve of mice transplanted with 0.8 million cells from the control sample, primary porcine hepatocytes (PPH) sample and hepatocyte maintenance medium (HMM) samples. (B) Immunohistochemical results using FAH antibody in livers of PPH‐FRG, HMM‐FRG and Control‐FRG mice 3 months after transplantation. (C) Immunostaining of FAH and porcine albumin in FRGN mice transplanted PPH and the cells treated with HMM and DMSO. (D) Porcine serum albumin levels in PPH‐FRG, HMM‐FRG and Control‐FRG mice 3 months after transplantation.

Given that cell repopulation is a key indicator of regeneration, which is a fundamental characteristic of primary hepatocytes, we conducted a transplantation experiment. We transplanted 0.8 million cells from each group into Fah‐deficient Rag2^−/−^Il2rg^−/−^NOD (FRGN) mice to assess the repopulation ability of the cells.^52^ Survival analysis revealed that mice receiving PPH and HMM‐treated cells had significantly improved survival rates compared to the control group treated with DMSO, with over 50% survival observed in the former groups (Figure 7A). Immunohistochemistry based on FAH staining was used to quantify repopulation efficiency (Figure 7B). Immunostaining of ALB further validated the quality of repopulated cells (Figure 7C). Besides, the secretion of porcine ALB in the blood of mice transplanted with HMM‐cell performs much better than that of the control group (Figure 7D), indicating that those repopulated cells could perform the hepatic functions. The results indicated that HMM‐treated cells possessed repopulation capability as that of PPH.

## 4 DISCUSSION AND CONCLUSION

Porcine hepatocytes are increasingly recognized as valuable substitutes for human primary hepatocytes in biomedical research and clinical applications due to their metabolic similarities. Yet, hepatocytes often dedifferentiate when cultured in vitro, losing their specialized functions and metabolic regulation observed in vivo. This led to inaccurate representations of hepatocyte characteristics. To tackle this challenge, our research team conducted the first single‐cell multi‐omics study of hepatocyte dedifferentiation, aiming to provide data resources to unravel the intricate process of cellular dedifferentiation and provide valuable clues for the hepatocyte's maintenance in vitro.

In our study, we initially utilized 10× multiome technology for sample preparation. However, we encountered challenges with lysing porcine hepatocytes. To overcome this hurdle, we systematically increased the concentration of NP40, a key reagent recommended in the 10× protocol for cell lysis. After several trials, we determined that doubling the NP40 concentration to 0.2% produced satisfactory results. In fact, this adaptation not only improved the efficiency of hepatocyte lysis but also broadened the utility of 10× multiome technology for processing solid tissues.

Compared to typical cell annotation, analysing rapidly changing samples for the single‐cell analysis presented more difficulties. In our study, hepatic markers for the cells undergoing dedifferentiation are indistinct^53^ and typical markers could not be directly employed. To overcome this obstacle, we adopted a strategy of first enriching specific pathways and then selecting pathway‐representative genes as markers. This method yielded relatively precise features for identifying cell populations, significantly advancing our understanding of cellular dedifferentiation processes.

Through comparative studies across species, we discovered that, unlike mouse hepatocytes which directly lose their identity, PPH can reacquire some hepatic characteristics under certain conditions (Figure 3H). When the cell density of PPH was low and cell contraction was lacking, the cells immediately proliferated and lost their hepatic functions. However, as the cell density increased and cell contraction strengthened, cell proliferation slowed down or even ceased. Interestingly, during this process, the cells reacquired some hepatocyte features. This observation may provide new insights for optimizing hepatocyte culture in the future.

After bioinformatic analysis, we identified and validated a series of related pathways, such as ERK, Src and TGF‐β. As some of them were reported in the hepatic dedifferentiation of other species, our findings further validated their conversation among different species. Notably, we identified that PI3K was involved in PPH dedifferentiation, and GDC0032 could significantly block PPH dedifferentiation. In addition to the insights gained from single‐cell analysis, we observed that aggregated culture conditions offered benefits in maintaining hepatocyte function during our experiments. Initially, when we evaluated the dedifferentiation process through RT‐qPCR, we noticed that samples from Day 7 began to upregulate partial hepatic genes (Figure S1A). This observation provided early indications of the potential advantages of cell aggregation in preserving hepatocyte characteristics. Single‐cell analysis of D7 samples further confirmed that certain clusters exhibited partial hepatic features (Figure 3D). In addition, immunostaining images and BODIPY 493/503 staining further revealed that cells expressing high levels of HNF4α and synthesizing lipid droplets were predominantly located at the center of high‐density area (Figure S1B,C, indicated with arrows). These clusters appeared only after cells had grown and reached confluence, typically observed around Day 7. Based on these findings, we hypothesized that cell aggregation could enhance hepatic functions and serve as a potential condition for preventing dedifferentiation. To validate this hypothesis, we conducted further experiments and, as expected, cell aggregation successfully blocked hepatocyte dedifferentiation (Figure 5A,B). Based on these findings, we further establish a combination to maintain PPH. It was shown that hepatocytes cultured with this combination can achieve better cellular functions.

Cell repopulation is a crucial manifestation of hepatic regeneration ability and is considered as the ultimate assay to evaluate hepatocyte function. FRG mouse model has been widely employed to identify the repopulation ability of hepatocytes. In this study, we transplanted primary hepatocytes treated with DMSO (negative control) and HMM for 5 days, as well as freshly isolated PPHs, into FRG mice. The repopulation ability was comprehensively evaluated based on mouse survival rates, cell chimerism percentages and the quality of chimeric cells. These results convincingly demonstrated that primary hepatocytes maintained with HMM retain their repopulation ability as well as that of PPH.

In conclusion, employing cutting‐edge techniques and in‐depth analyses, we provided a comprehensive description of hepatocyte dedifferentiation at the single‐cell level. Based on bioinformatic analysis, we explored and validated a series of crucial targets, which enabled us to develop a chemical combination to inhibit hepatic dedifferentiation in vitro, yielding encouraging results. This breakthrough introduces new strategies for preserving porcine hepatocyte function ex vivo and offers insights into addressing dedifferentiation in hepatocytes from other species.

## AUTHOR CONTRIBUTIONS

Conceptualization: JH and MS. Methodology: MS, JH, ZW, JR and SC. Investigation: MS, JH, ZW, JR, SC, JCL, ZX and ZH. Formal analysis: ZW, JH, MS, SC, JCL and JL. Validation: JH, JR, JY and WD. Visualization: JH, ZW, JR, SC, JY and WD. Writing: MS, JH, ZW and MZ. Funding acquisition: MS, MZ, SD, ZW, YY and HT. Resources: MZ, SD, YY, TH and ZW. All authors read and approved the final manuscript.

## FUNDING INFORMATION

This work was supported by the National Key R&D Program of China (2018YFA0801402) to MLS. China National Postdoctoral Program for Innovative Talents (BX20230229), China Postdoctoral Science Foundation (2024M752038) to ZYW. National Key R&D Program of China (2023YFF1204500), National Natural Science Foundation of China (62103262) to YY. National Key R&D Program of China (2017YFA0104001) and the National Natural Science Foundation of China (32030031 and 31530025) to SD. MQZ is supported by the Cecil H. and Ida Green Distinguished Chair. National Key Research and Development Program 2023YFC3404305. CAS Project for Young Scientists in Basic Research (YSBR‐012).

## CONFLICT OF INTEREST STATEMENT

All co‐authors have consented to this submission, and there are no conflicts of interest to declare.

## Supporting information


**Data S1:** Supplementary figures.

**Table S1.** The counts and proportion of cells sampled on different days within each cell cluster.

**Table S2.** The AUC (Area Under of Curve) value of each regulon in each cluster.

## Data Availability

The raw sequence data reported in this paper have been deposited in the Genome Sequence Archive^54^ in the National Genomics Data Center,^55^ China National Center for Bioinformation/Beijing Institute of Genomics, Chinese Academy of Sciences (GSA: CRA014896) that are publicly accessible at https://ngdc.cncb.ac.cn/gsa.
