# Supplementary material for: Single‐cell multi‐omics deciphers hepatocyte dedifferentiation and illuminates maintenance strategies
Source: Cell Prolif. 2025 Jan 14;58(3):e13772. doi: 10.1111/cpr.13772 (PMC11882756; doi:10.1111/cpr.13772)
Supplement: Supplementary file 1 — Data S1: Supplementary figures. [file CPR-58-e13772-s003.pdf]

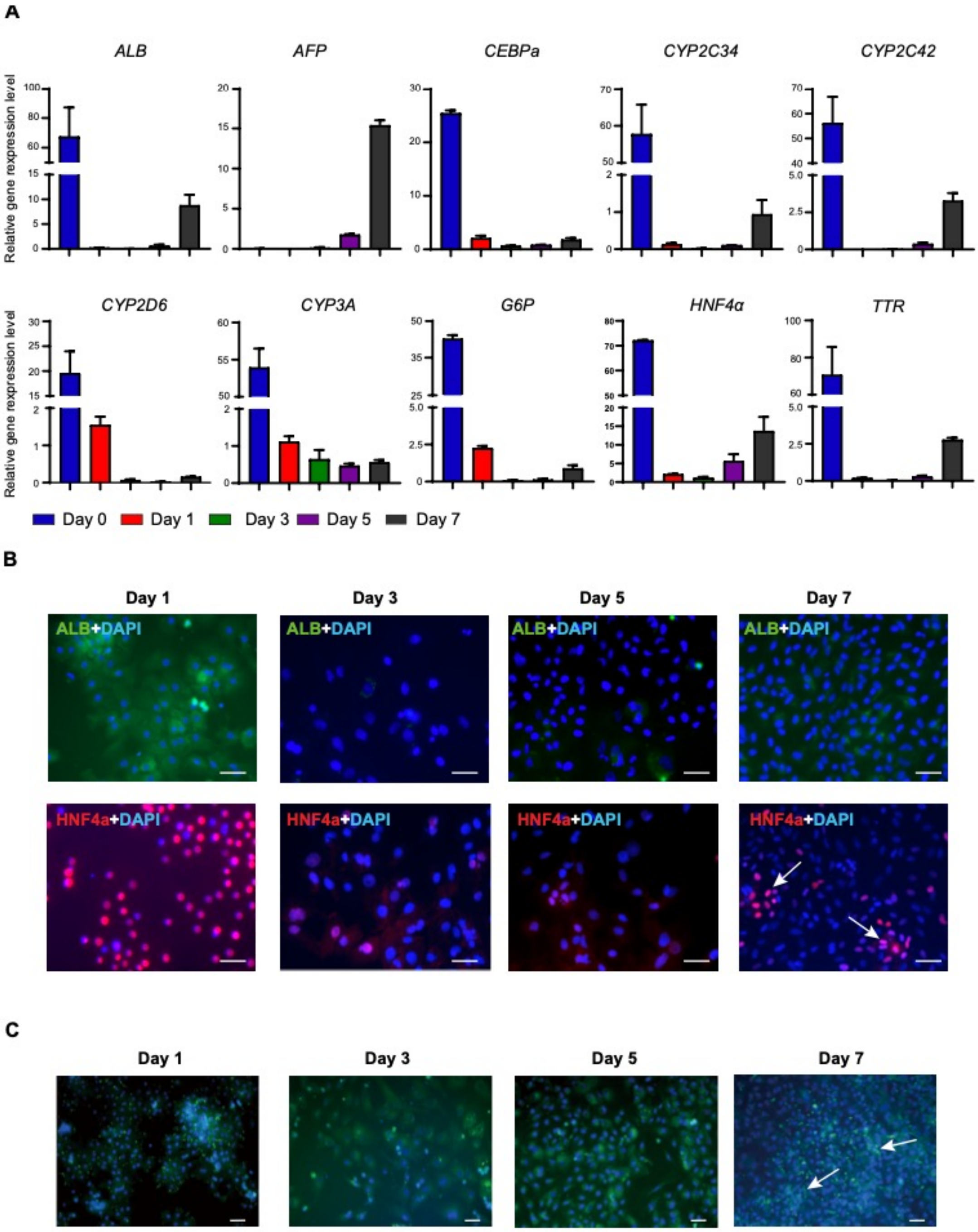

Figure S1. Caption of PPH dedifferentiation

A. The mRNA expression level of hepatic genes in hepatocytes at Day1, Day3, Day5 and Day7 after isolation.

B. The Immunostaining of ALB and HNF4α in cultured hepatocytes at Day1, Day3, Day5 and Day7 after isolation. Arrow indicated the high density area. Scale bars, 50 μm.

C. The BODIPY 493/503 staining of cultured hepatocyte at Day1, Day3, Day5 and Day7 after isolation. Arrow indicated the high density area. Scale bars, 50 μm.

A

			Day 0	Day 1	Day 3	Day 5	Day 7
Gene expression	Sequencing	Sequenced read pairs	359,311,303	423,552,232	351,445,303	295,203,905	332,936,786
		Valid barcodes	91.90%	92.50%	95.00%	92.40%	92.40%
		Percent duplicates	45.60%	62.50%	49.50%	43.70%	52.80%
	Cells	Estimated number of cells	12,187	6,757	7,739	7,841	6,385
		Median genes per cell	2,156	2,534	3,589	2,996	3,085
		Total genes detected	24,377	25,712	25,291	24,736	24,547
	Targeting	Reads mapped to genome	93.00%	95.90%	95.50%	96.50%	96.80%
		Reads mapped confidently to genome	72.10%	56.80%	80.70%	79.70%	81.60%
ATAC	Sequencing	Sequenced read pairs	185,650,828	167,817,729	141,959,538	144,370,627	117,438,319
		Valid barcodes	96.30%	97.30%	97.30%	97.90%	94.80%
		Percent duplicates	12.70%	13.50%	6.40%	6.60%	10.50%
	Cells	Estimated number of cells	12,187	6,757	7,739	7,841	6,385
		Fraction of high-quality fragments in cells	77.80%	57.40%	77.20%	86.70%	83.90%
		Fraction of transposition events in peaks in cells	51.90%	28.40%	38.40%	48.10%	48.40%
	Targeting	Number of peaks	132,641	89,099	125,117	152,314	154,633
		Fraction of genome in peaks	4.70%	3.20%	4.30%	5.20%	5.30%
		TSS enrichment score	32.19	17.7	9.72	10.57	8.44
	Mapping	Confidently mapped read pairs	79.50%	83.80%	87.00%	86.10%	88.00%

B

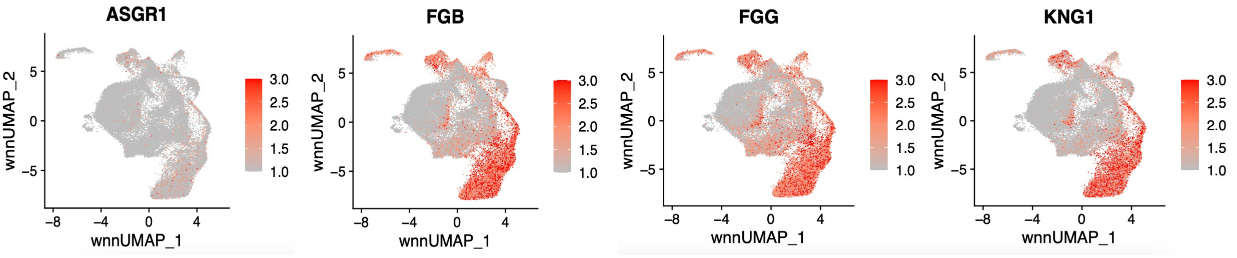

Figure S2. Quality control and expression distribution of several hepatic genes

A. Quality control of RNA/ATAC data.
B. UMAP shows the expression distribution hepatic genes that reappear on D7.

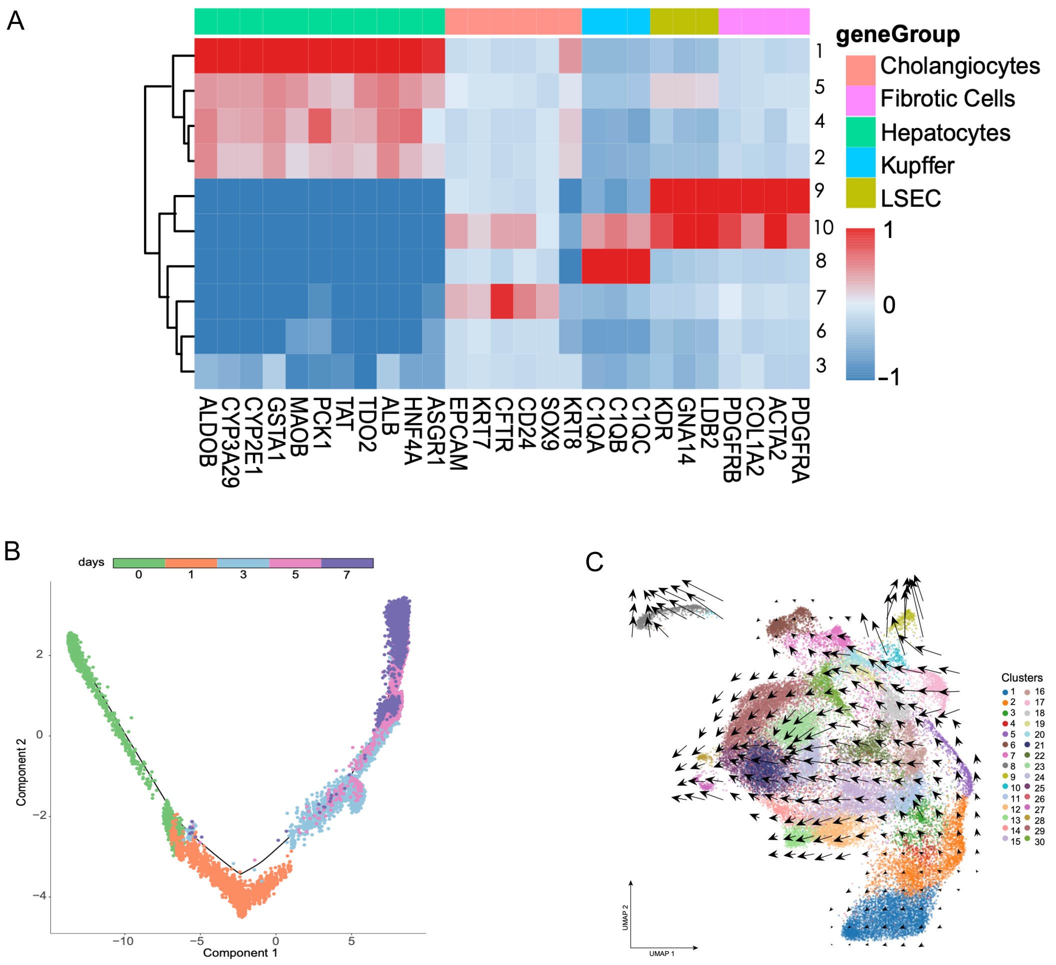

Figure S3. D0 cell annotation and cell trajectory among cells from D0 to D7.

A. Heatmap showing scaled mean expression of known marker genes in D0 cluster.
B. Monocle2 analysis showing the cell trajectory among cells from D0 to D7.
C. scVelo analysis showing the direction of cell differentiation among cell clusters.

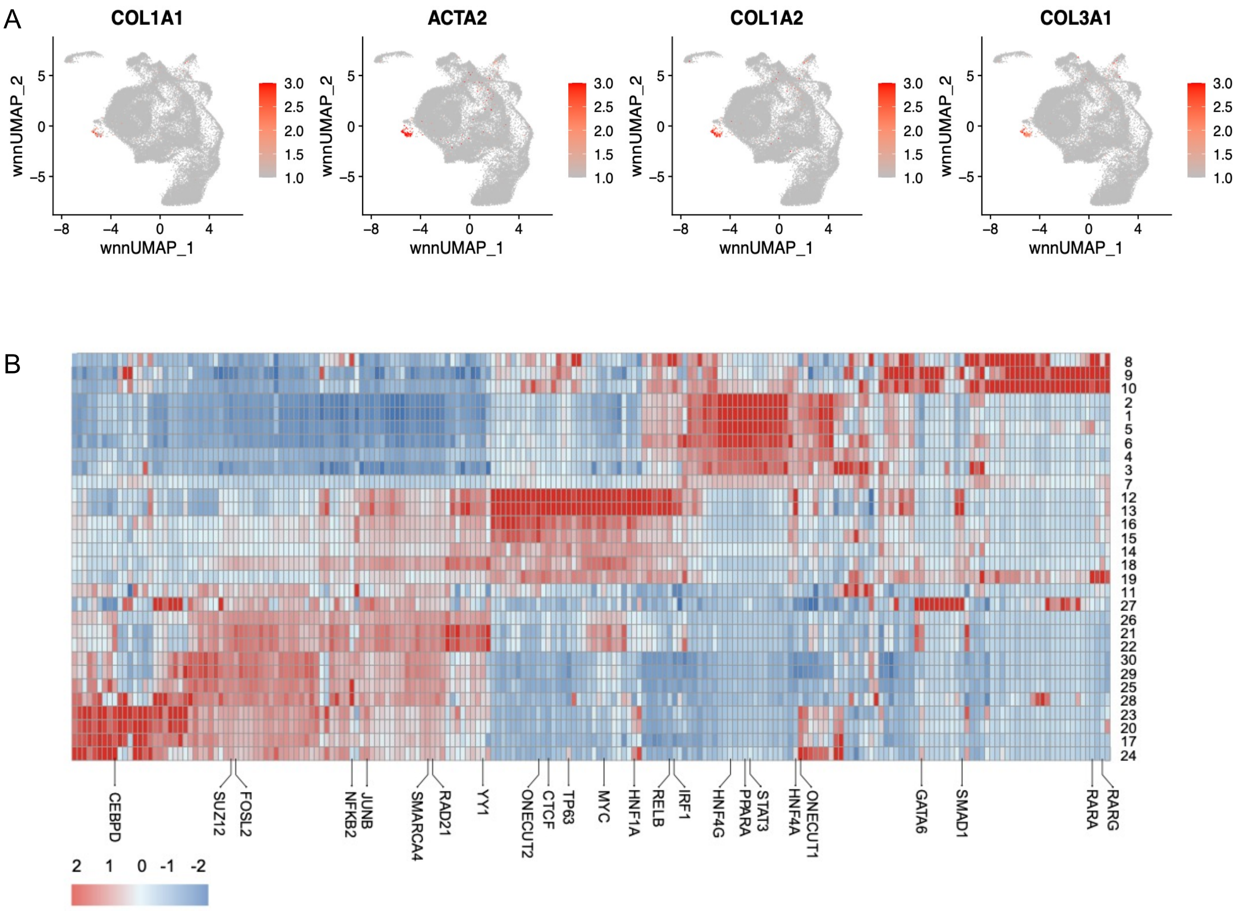

Figure S4. TF regulon of each cell cluster and expression distribution of collagen-related genes.

A. UMAP shows the expression distribution of collagen-related genes.

B. TF regulon of each cell cluster.

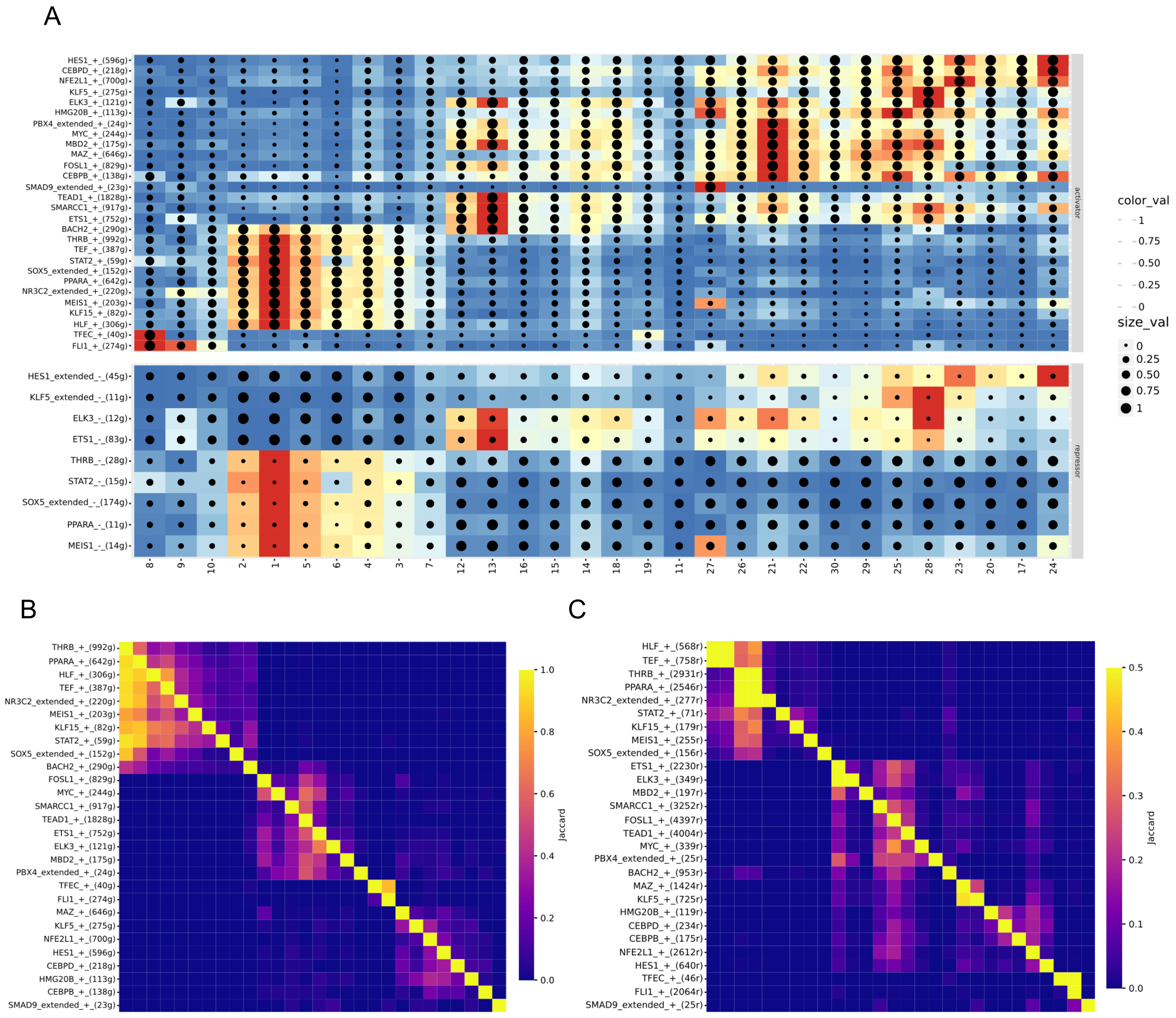

Figure S5 eGRN analysis by SCENIC+ algorithm

A. SCENIC+ eGRN enrichment dot plot. The gene-based eRegulon specificity score (RSS) in the cell type is shown by circle size, and the colour represents the TF expression in the corresponding cell type. Cell clusters are ordered on the basis of their gene expression similarity. The symbol in the right column indicates whether the TF activates (+) or represses (-) its target genes.
B. Overlap of target regions of eRegulons. The overlap is divided by the number of target regions of the eRegulon in each row. fr., fraction
C. Overlap of target genes of eRegulons. The overlap is divided by the number of target genes of the eRegulon in each row.

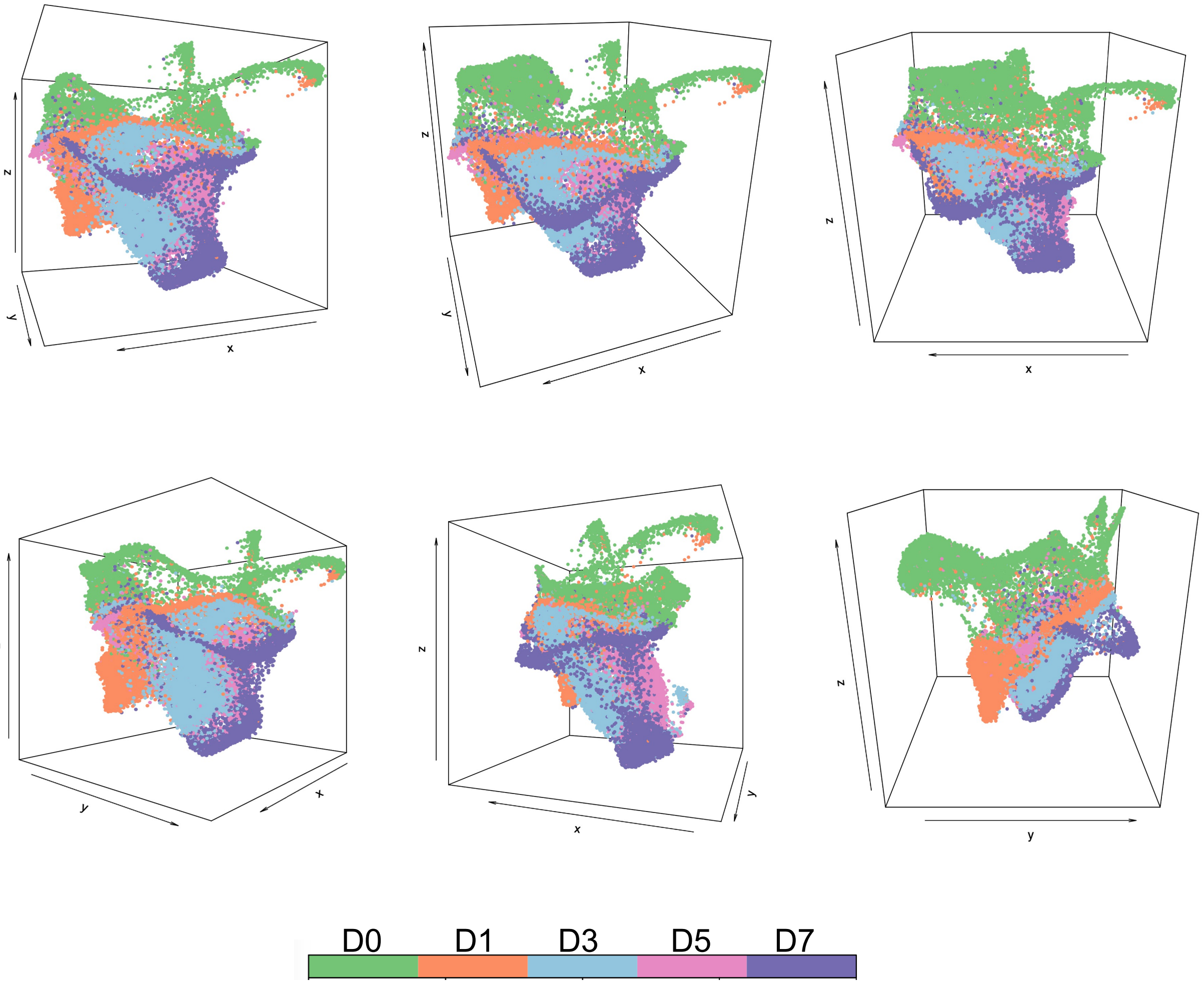

Figure S6 The three-dimensional spatial distribution of cells on different days in UMAP.

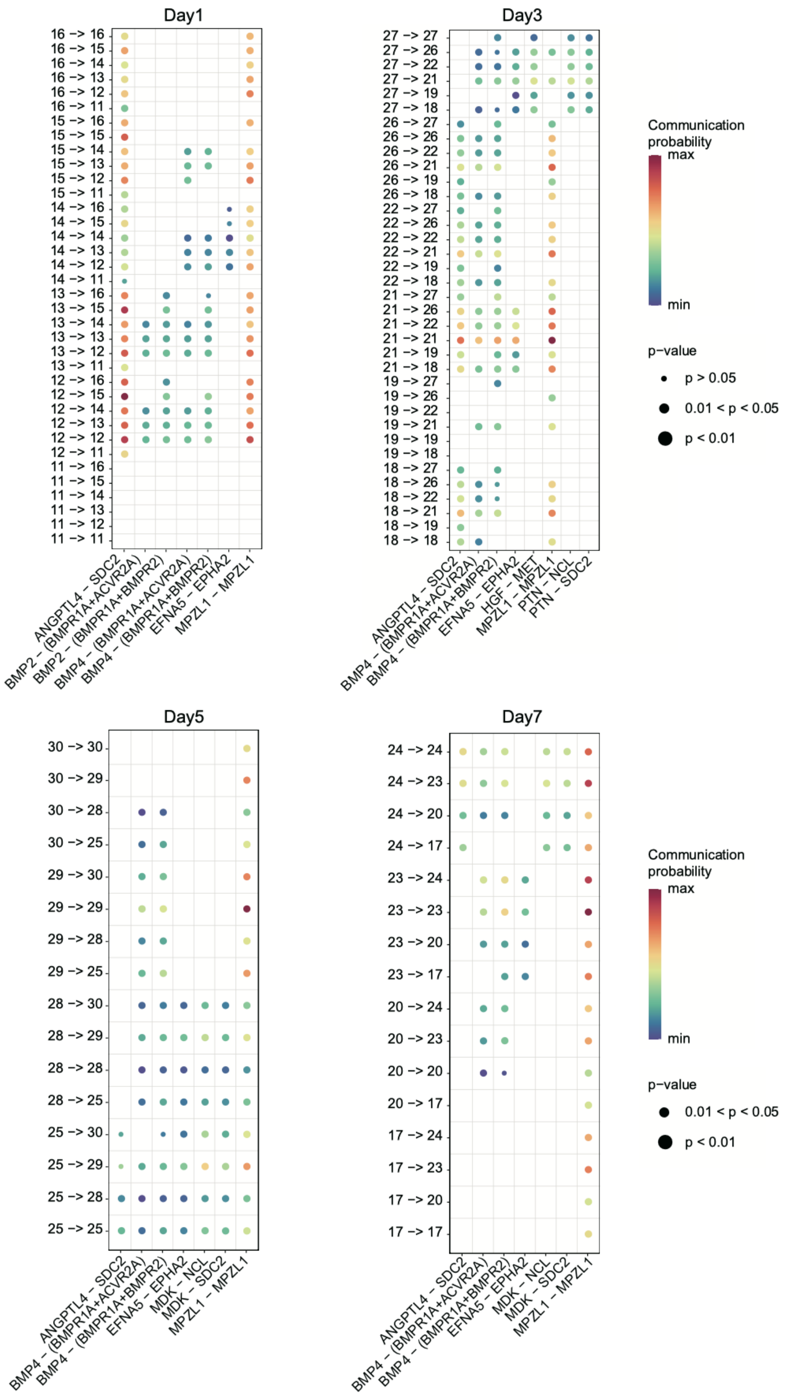

Figure S7 Ligand-receptor pairs enriched in D1, D3, D5, D7 cell clusters, respectively.

Target	Related signaling pathway	Related Compound	Cas Number	Structure
TGIF1	TGF-beta pathway	A83-01	909910-43-6	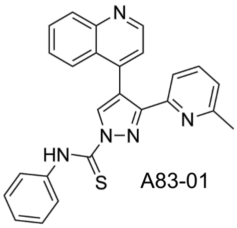 A83-01
TGFBR1	TGF-beta pathway	A83-01	909910-43-6	
SMAD4	TGF-beta pathway	A83-01	909910-43-6	
SRC	SRC	SB203580	152121-47-6	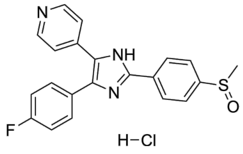 SB203580
PIK3R1	PI3K	GDC0032	1282512-48-4	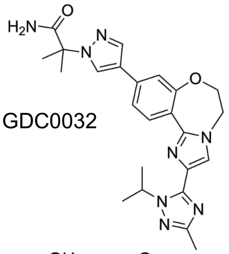 GDC0032
DUSP4	ERK/MAPK	PD0325901	391210-10-9	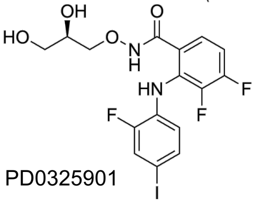 PD0325901
SHC3	ERK/MAPK	PD0325901	391210-10-9	
MAPK1	ERK/MAPK	PD0325901	391210-10-9	
MAPKAPK2	ERK/MAPK	PD0325901	391210-10-9	
MAPKAPK3	ERK/MAPK	PD0325901	391210-10-9	

Figure S8 chemical inhibitors used to suppress the dedifferentiation of hepatocytes based on signaling pathways activated on D1
